# Possible Role of Matrix Metalloproteinases and TGF-β in COVID-19 Severity and Sequelae

**DOI:** 10.1089/jir.2021.0222

**Published:** 2022-08-18

**Authors:** Gustavo Ramírez-Martínez, Luis Armando Jiménez-Álvarez, Alfredo Cruz-Lagunas, Sergio Ignacio-Cortés, Itzel Alejandra Gómez-García, Tatiana Sofia Rodríguez-Reyna, José Alberto Choreño-Parra, Joaquín Zúñiga

**Affiliations:** ^1^ Laboratory of Immunobiology and Genetics, Instituto Nacional de Enfermedades Respiratorias “Ismael Cosío Villegas,” Mexico City, Mexico.; ^2^ Escuela de Medicina y Ciencias de la Salud, Tecnológico de Monterrey, Mexico City, Mexico.; ^3^ Department of Immunology and Rheumatology, Instituto Nacional de Ciencias Médicas y Nutrición Salvador Zubirán, Mexico City, Mexico.

**Keywords:** SARS-CoV-2, COVID-19, MMPs, TGF-β, ARDS, pulmonary fibrosis

## Abstract

The costs of coronavirus disease 2019 (COVID-19) are devastating. With millions of deaths worldwide, specific serological biomarkers, antiviral agents, and novel therapies are urgently required to reduce the disease burden. For these purposes, a profound understanding of the pathobiology of COVID-19 is mandatory. Notably, the study of immunity against other respiratory infections has generated reference knowledge to comprehend the paradox of the COVID-19 pathogenesis. Past studies point to a complex interplay between cytokines and other factors mediating wound healing and extracellular matrix (ECM) remodeling that results in exacerbated inflammation, tissue injury, severe manifestations, and a sequela of respiratory infections. This review provides an overview of the immunological process elicited after severe acute respiratory syndrome coronavirus-2 (SARS-CoV-2) infection. Also, we analyzed available data about the participation of matrix metalloproteinases (MMPs) and transforming growth factor-beta (TGF-β) in immune responses of the lungs. Furthermore, we discuss their possible implications in severe COVID-19 and sequela, including pulmonary fibrosis, and remark on the potential of these molecules as biomarkers for diagnosis, prognosis, and treatment of convalescent COVID-19 patients. Our review provides a theoretical framework for future research aimed to discover molecular hallmarks that, combined with clinical features, could serve as therapeutic targets and reliable biomarkers of the different clinical forms of COVID-19, including convalescence.

**Figure:**
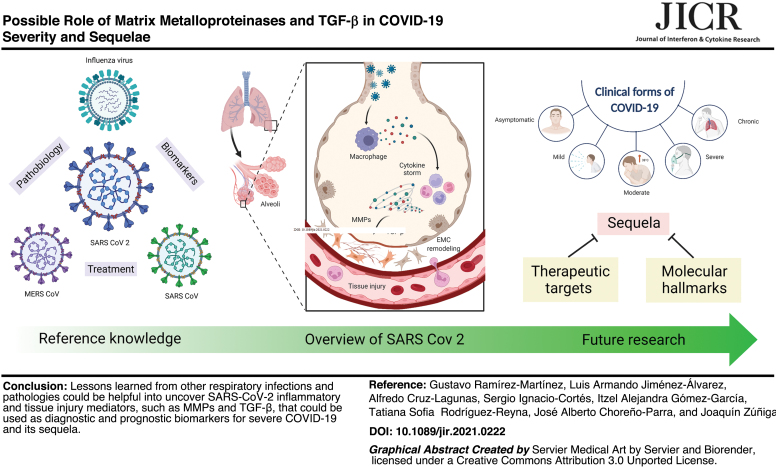
Color images are available online.

## Introduction

The coronavirus disease 2019 (COVID-19), caused by the severe acute respiratory syndrome coronavirus-2 (SARS-CoV-2), is now the leading infectious cause of death worldwide. In the United States, as of October 2020, COVID-19 was the second and third cause of death in persons older than 85 and 45 years, respectively (Woolf and others 2021). The most recent global report estimates that until July 2021, 186,240,393 infections and 4,027,861 deaths have occurred around the world (Johns Hopkins Coronavirus Resource Center 2022). The current pandemic could extend for additional months due to the lack of sufficient vaccines to immunize a massive proportion of the world population in developing countries. The absence of specific antiviral drugs is an additional hurdle that limits our capacity to prevent poor clinical outcomes and reduce the mortality of COVID-19 patients. This crisis could be further aggravated by the emergence of novel SARS-CoV-2 strains with increased transmission and pathogenic potential.

Over the past year, major advances in our knowledge about the pathogenesis of COVID-19 have been made. However, the current understanding of the host and pathogen factors determining the clinical behavior of the disease is still incomplete. Identifying molecular hallmarks associated with severe disease that could serve as prognostic biomarkers is relevant to guide therapeutic decisions. Furthermore, studying immune parameters of severity could reveal targets for the development of novel immune therapies. Importantly, an increasing incidence of sequelae has been observed in convalescent COVID-19 patients, including pulmonary fibrosis (PF), especially among those who recovered from severe disease. These complications could permanently affect the respiratory function of patients impacting negatively on their quality of life. Thus, a higher interest in pathogenic processes underlying excessive inflammation, tissue injury, and extracellular matrix (ECM) remodeling after SARS-CoV-2 infection is urgent.

Unfortunately, little literature exists on the causative mechanisms of post-COVID-19 PF. Hence, in the current review, we summarize available data generated from other respiratory infectious diseases, focusing on the role of matrix metalloproteinases (MMPs) and the transforming growth factor-beta (TGF-β) as immune mediators and ECM remodelers in the lungs. Our goal is to provide a theoretical framework that could serve as a reference for future investigations looking for diagnostic and prognostic biomarkers, as well as therapeutic targets to prevent severe disease and sequelae in COVID-19 patients.

## Clinical and Immunological Features of COVID-19

SARS-CoV-2 is an enveloped, positive-sense, single-stranded RNA virus belonging to the *Coronaviridae* family, with a genome of 29.9 kb. As a zoonotic virus, SARS-CoV-2 is known to have bats and pangolins as its main reservoirs. Indeed, coronaviruses isolated from both species share 90.55% and 91.02% of genome identity with SARS-CoV-2, respectively (Konda and others 2020). After being inhaled, SARS-CoV-2 reaches the host nasal cavity and enters into the epithelial cells by attaching to the angiotensin-converting enzyme 2 (ACE2) receptor through its envelope spike (S) protein (de Wit and others 2016; Shang and others 2020). The virus is then internalized into endosomes, and finally, the viral and lysosomal membranes get fused (Li 2016; Shang and others 2020).

Compared with SARS-CoV, the receptor-binding domain (RBD) of the S protein of SARS-CoV-2 has a higher affinity for ACE2 and is less exposed to the host cell immune surveillance. Another interesting feature of SARS-CoV-2 is that its S protein is preactivated before cell entry by the proprotein convertase furin, making the infection process independent of cell protease activity. Altogether, these factors favor a highly efficient infective capacity of SARS-CoV-2 (Shang and others 2020).

Once SARS-CoV-2 replicates in the upper airways, it disseminates early to the lower respiratory tract, where a more pronounced innate immune response occurs, leading to the onset of clinical manifestations. The most frequent symptoms are fever, cough, and fatigue, followed by headache, diarrhea, dyspnea, anosmia, and loss of taste (Huang and others 2020; Li and others 2020). Nonetheless, COVID-19 exhibits a heterogeneous clinical spectrum that includes asymptomatic cases and mild-to-severe manifestations, which develop respiratory failure, multiorgan dysfunction, and even death (Grasselli and others 2020; Wang and others 2020a). Individuals who progress to severe COVID-19 exhibit pneumonia within 10–20 days after symptoms onset, associated with reduced oxygen saturation, acute respiratory distress syndrome (ARDS), and prominent lung damage with ground-glass opacities.

Among other clinical features in severe COVID-19 patients are lymphopenia, thrombocytopenia, and a raise of inflammatory markers such as C-reactive protein (CRP), increased D-dimer, lactate dehydrogenase (LDH), and proinflammatory cytokines (Costela-Ruiz and others 2020; Guan and others 2020; Huang and others 2020; Mason 2020; Tang and others 2020a). Intriguingly, although the majority of SARS-CoV-2-infected individuals exhibit respiratory manifestations, there are individuals who only present gastrointestinal symptoms, which include diarrhea, abdominal pain, vomiting, nausea, and anorexia (Perisetti and others 2020). Indeed, SARS-CoV-2 has been detected in stool samples of asymptomatic COVID-19 patients and it has been documented that SARS-CoV-2 can infect and replicate in gastrointestinal and liver cells. Indeed, ACE2 is expressed in gastrointestinal mucosa epithelial cells, where it replicates and can infect other tissues and organs that express ACE2, such as the liver (Sahu and others 2021).

Also, some authors have emphasized the changes in the gut microbiota (dysbiosis) that the SARS-CoV-2 infection can cause and the effect that this might have on health (Kaźmierczak-Siedlecka and others 2020; Villapol 2020). However, what can influence that some patients develop severe lung disease? Since the start of the pandemic, the most affected persons were individuals older than 65 years and individuals with comorbidities, who exhibit a reduced immune response. So it might be plausible that individuals with interferon deficiencies, elevated inflammatory markers, and decreased lymphocyte count, among others, could not contain and clear the viral infection and thus develop severe lung disease.

Clinical manifestations and severity of COVID-19 are also associated with age. As such, older adults with comorbidities show a higher risk for developing ARDS and mortality. Conversely, children rarely present with severe clinical manifestations, some resembling Kawasaki disease with cardiovascular involvement (Weisberg and others 2021). Recent data have shown that severe disease can also occur in younger patients with no preexisting medical conditions (Merad and Martin 2020).

The excessive release of proinflammatory mediators, termed the cytokine storm, is the proposed cause of ARDS among COVID-19 patients. Of note, other organs besides the lungs can also be affected, including the brain, heart, intestines, kidneys, and liver. This inflammatory reaction induces the production of several defense proteins such as CRP and ferritin in the liver, which further enhance inflammation and sustain the cytokine storm in severe COVID-19 patients (Ruscitti and others 2020; Meng and others 2021). In fact, increased levels of ferritin predict the risk of death in these patients (Ruscitti and others 2020). In addition, the hyperinflammation observed in critically ill COVID-19 patients admitted to the intensive care unit (ICU) causes thrombotic events.

Abnormal coagulation parameters such as higher D-dimer levels, longer prothrombin time, and activated partial thromboplastin time have been associated with poor prognosis. These abnormal coagulation parameters occur early after hospitalization, and in some patients, fibrinogen concentrations and antithrombin activity decreased over time (Becker 2020; Klok and others 2020; Tang and others 2020b).

## The Cytokine Storm Syndrome of Severe COVID-19

Approximately 15% of COVID-19 patients exhibit pneumonia, and 5% will turn into ARDS, septic shock, and organ failure (Cao 2020; Huang and others 2020). Whereas a typical host antiviral response involves the production of some proinflammatory cytokines and the activation of CD4^+^ and CD8^+^ T cells to control the infection, COVID-19 patients display an exacerbated cytokine production response with variable functionality of innate and adaptive lymphocytes. For instance, in some patients, the presence of interstitial mononuclear inflammatory infiltrates dominated by lymphocytes in the lungs is accompanied by the overactivation of peripheral blood Th17 and cytotoxic CD8 T cells (Xu and others 2020b).

Conversely, a group of severe COVID-19 patients have pronounced lymphopenia and depletion of helper and suppressor T cells, B cells, and natural killer (NK) cells from the blood, perhaps as a consequence of direct effects by the virus (Qin and others 2020; Wang and others 2020b). This could be explained by the inability of these individuals to control the viral infection, leading to an uncontrolled immune response and thus an overproduction of cytokines. In fact, the persistent T cell stimulation, along with a chronic inflammatory process, conduces to T cell exhaustion and consequently lymphopenia (Fathi and Rezaei 2020).

Diao and others reported a drastic reduction of CD4^+^ and CD8^+^ T cells, particularly in COVID-19 patients admitted to the ICU. Moreover, both T cell subpopulations exhibited an increase in cell surface expression of programmed cell death protein 1 (PD-1) and T cell immunoglobulin and mucin domain-containing-3 (Tim-3), which confirmed T cell exhaustion (Diao and others 2019).

ARDS is characterized by increased lung permeability, severe hypoxemia, and noncardiogenic pulmonary edema. These conditions disrupt the alveolar-capillary barrier and result from systemic hyperinflammation (Bernard and others 1994; Cabrera-Benitez and others 2014; Guillamat-Prats and others 2017). The cytokine storm syndrome of severe COVID-19 is characterized by high circulating levels of interleukin-2 (IL-2), interleukin-6 (IL-6), interleukin-7 (IL-7), interleukin-10 (IL-10), interleukin-17 (IL-17), granulocyte-macrophage colony-stimulating factor (GM-CSF), C-X-C motif chemokine ligand 10 (CXCL10), C-C motif chemokine ligand 2 (CCL2), C-C motif chemokine ligand 3 (CCL3), and tumor necrosis factor (TNF) (Table 1) (Huang and others 2020). Indeed, this exuberant immune activation predicts poor prognosis in COVID-19 and resembles other cytokine release syndromes such as the macrophage activation syndrome (Merad and Martin 2020; Tang and others 2020c).

**Table 1.** Main Cytokines Involved in the Cytokine Storm in Severe COVID-19

Cytokine	Role in severe COVID-19	Possible role in COVID-19 sequela	References
IL-1β	Induced in monocytes/macrophages upon lung epithelial damage by SARS-CoV-2. IL-1β induce IL-1 for the recruitment and activation of innate immune cells.	Maintains a persistent inflammasome activation that might lead to organ damage.	van de Veerdonk and Netea (2020)
IL-2	Produced by activated CD4^+^ T cells and CD8^+^ T cells. Enhances CD4^+^ and CD8^+^ T cell activation by stimulating expansion and differentiation of T cells.	Possible impairment of lung T cell pool to contain the viral infection.	de Bree and others (2005); Wilson and Livingstone (2008); Abbas and others (2018); Shi and others (2020a)
IL-4	Essential cytokine of the Th2 immune response.	Induces the activation of M2 macrophages, realizing TGF-β and platelet-derived factor, thus promoting the expansion of resident fibroblasts and the formation of a temporary matrix.	Diao and others (2019); Vaz de Paula and others (2020)
IL-5	Eosinophil-stimulating cytokine. Attracts eosinophils in respiratory tissues.	Recruitment of eosinophils to the lung, where they contribute to trigger an impaired immune response.	Gorski and others (2019); Pala and Pistis (2021)
IL-6	Proinflammatory cytokine. Clinically associated with prognosis.	Involved in COVID-19-associated coagulopathy, stimulates platelet activity, and induces endothelial dysfunction.	Levi and van der Poll (2005); Du and others (2021); Potere and others (2021)
IL-7	IL-7/IL-7R imbalance leads to lymphopenia.	Contributes to the loss of naive T cells impairing the antiviral activity.	Bekele and others (2021)
IL-10	Proinflammatory and immune-activating role.	Participates in T cell exhaustion by overactivation and proliferation. Hyperactivation of the adaptive immune response that exacerbates the disease.	Diao and others (2019); Lu and others (2021)
IL-12	Enhances cytotoxic activity of NK cells. It is a key inducer of Th1 cell differentiation.	Contributes to tissue damage.	
IL-13	Essential cytokine of the Th2 immune response. Contributes to lung inflammation during COVID-19.	Promotes alternatively activated macrophages, which contribute to long-term lung pathology.	Vaz de Paula and others (2020); Donlan and others (2021)
IL-17	Triggers the production of other proinflammatory cytokines, such as IL-6.	Impairs neutrophil recruitment, stimulates proinflammatory mediators, and prevents apoptosis.	Maione and others (2021)
GM-CSF	A later and excessive inflammatory response that perpetuates the cytokine storm.	Induces CD14^+^ and CD16^+^ monocytes, which release IL-6 and GM-CSF, worsening the cytokine storm in the lung.	Bonaventura and others (2020); Zhou and others (2020)
CXCL10	CXCL10^+^ CCL2^+^ inflammatory macrophages are predominant in BAL.	Contributes to lung hyperinflammation and tissue damage.	Zhang and others (2021)
CCL2	CXCL10^+^ CCL2^+^ inflammatory macrophages are predominant in BAL.	Contributes to lung hyperinflammation and tissue damage.	Zhang and others (2021)
CCL3	Increased in serum.	Promotes inflammation and tissue damage in the lung.	Xu and others (2020c)
CCL11	Proinflammatory activity.	Promotes inflammation and tissue damage in the lung.	Xu and others (2020c); Choreño-Parra and others (2021)
TNF	Activates inflammatory CXCL10^+^ CCL2^+^ macrophages.	Synergism with IFN-γ to drive inflammatory macrophage phenotype in lung and tissue inflammation and damage.	Zhang and others (2021)
VEGF	Positively correlates with sequential organ failure assessment.	Increases vascular permeability, alters the homeostasis of endolethial cells, produces blood clots.	Kong and others (2020); Choreño-Parra and others (2021)
TWEAK	Involved in the aberrant immune response.	Stimulates lung inflammation.	Yalçın Kehribar and others (2020); Choreño-Parra and others (2021)
TSLP	Essential cytokine of the Th2 immune response.	Enhances lung inflammation.	Toki and others (2020); Choreño-Parra and others (2021)
MMP-1	Involved in tissue damage.	Inducing PF. Involved in reduced mitochondrial function, increases of HIF-1α expression, diminished production of ROS, and promotion of a proliferative–migratory–antiapoptotic phenotype in alveolar epithelial cells.	Herrera and others (2013); Choreño-Parra and others (2021)
MMP-3	Involved in tissue damage.	Considered a proinflammatory factor inside several organs and linked to tissue damage. Initiates the degradation process of ECM.	Choreño-Parra and others (2021); Guizani and others (2021); Wan and others (2021)

BAL, bronchoalveolar lavage; CCL, C-C motif chemokine ligand; COVID-19, coronavirus disease 2019; CXCL10, C-X-C motif chemokine ligand 10; ECM, extracellular matrix; GM-CSF, granulocyte-macrophage colony-stimulating factor; IFN-γ, interferon-gamma; IL, interleukin; MMP, matrix metalloproteinase; NK, natural killer; PF, pulmonary fibrosis; ROS, reactive oxygen species; SARS-CoV-2, severe acute respiratory syndrome coronavirus-2; TGF-β, transforming growth factor beta; TNF, tumor necrosis factor; TSLP, thymic stromal lymphopoietin; TWEAK, tumor necrosis factor-like weak inducer of apoptosis; VEGF, vascular endothelial growth factor.

Nevertheless, evidence suggests that IL-6, IL-10, and CXCL10 are the cytokines that correlate the most with disease progression. In particular, CXCL10 exhibits a persistent elevated expression pattern in COVID-19 patients, while in patients with other viral infections only a transient expression is observed (Buszko and others 2021). While the role of IL-6 and IL-10 in severe COVID-19 patients has been related to the acceleration of the inflammatory process and the induction of the cytokine storm, the role of CXCL10 has been associated with the recruitment of leukocytes to inflamed tissues, thus perpetuating inflammation and causing finally tissue damage (Costela-Ruiz and others 2020).

Moreover, the immunological profile of severe COVID-19 has unique features that differentiate the disease from other respiratory infections, such as the pandemic influenza A (H1N1). Among the immune factors found only in critical ill COVID-19, but not influenza, patients are interferon-gamma (IFN-γ), IL-4, IL-5, IL-6, IL-10, IL-12, IL-13, IL-1β, C-C motif chemokine ligand 11 (CCL11), vascular endothelial growth factor (VEGF), tumor necrosis factor-like weak inducer of apoptosis (TWEAK), thymic stromal lymphopoietin (TSLP), MMP-1, and MMP-3 (Choreño-Parra and others 2021). These molecules could play a specific role in COVID-19 and are potential targets to reduce its morbidity and mortality.

A key to understanding how soluble immune mediators lead to severe disease and subsequent chronic complications of viral infections is to analyze the interplay of specific cytokines, their properties, and effects on antiviral immunity, cell function, and tissue repair during and after infection. For instance, among the spectrum of cytokines elevated in critically ill COVID-19 patients, IL-6 is a pleiotropic molecule regulating a significant number of genes and having a dual role in antiviral cellular responses. In murine models of infection with influenza, vaccinia virus (VACV), vesicular stomatitis virus (VSV), and hepatitis B virus (HBV), IL-6 is essential to mount a protective immune response (Kopf and others 1994; Kuo and others 2009; Harker and others 2011; Lauder and others 2013; Bouezzedine and others 2015; Yang and others 2017).

Conversely, in other clinical studies and *in vitro* assays using human and murine cells, this cytokine is linked to viral persistence and worse clinical outcomes (Hou and others 2014; Wu and others 2015; Bardhan and others 2016). Interestingly, IL-6 is pivotal in the immune microenvironment that mediates persistent inflammation, lung tissue damage, and subsequent fibrosis, but also plays a bidirectional role since its early upregulation in mice with lung injury and PF caused by bleomycin (BLM) has antifibrotic effects by regulating the cell fate of type 2 pneumocytes (Kobayashi and others 2015). These data suggest that spatiotemporal factors might determine the protective or pathogenic properties of IL-6 during respiratory infections, which warrant further investigation in COVID-19.

Another immune signature detected in critically ill patients with COVID-19 includes the type 2 cytokines IL-4, IL-5, IL-13, and TSLP, related to noninfectious lung diseases (Choreño-Parra and others 2021). These cytokines participate in processes that cause repeated cycles of epithelial injury and immune activation in severe asthma patients, leading to chronic lung inflammation and fibrosis (Gubernatorova and others 2021). Hence, the immune mechanisms of lung injury that operate during severe COVID-19 might resemble certain aspects of the pathobiology of allergic disorders of the lung. Thus, addressing the role of other cytokines involved in allergic lung inflammation might help identify pathogenic factors in COVID-19. For instance, TSLP is an alarmin secreted during allergen-induced lung epithelial damage that favors eosinophilic inflammation in asthma by promoting the release of effector Th2 cytokines such as IL-4, IL-5, and IL-13 by innate and adaptive immune cells (Beckert and others 2020).

Other alarmins that can trigger type 2 inflammation, such as IL-25 and IL-33, as well as cytokines involved in inflammation and lung tissue repair such as IL-22, TNF, and TGF-β, have not been extensively addressed for their participation in morbidity and sequela of COVID-19 (Johnson and others 2013; Roan and others 2019; Wu and others 2019).

An additional factor that might contribute to disease severity in respiratory infections is related to the epithelial injury that triggers the release of damage-associated molecular patterns (DAMPs), which in turn activate “sterile inflammation” (Planté-Bordeneuve and others 2021). Likewise, during viral infection, the structural integrity of mucosal barriers can be disrupted, favoring translocation of pathogen-associated molecular patterns (PAMPs) beyond the respiratory epithelium. Together, both DAMPs and PAMPs turn on NFκB, MAPK, and interferon signaling pathways, perpetuating inflammation (Takeuchi and Akira 2010; Hiemstra and others 2015). These mechanisms must be extensively evaluated in COVID-19 to reveal further details about the immunopathology of severe manifestations.

**FIG. 1.**
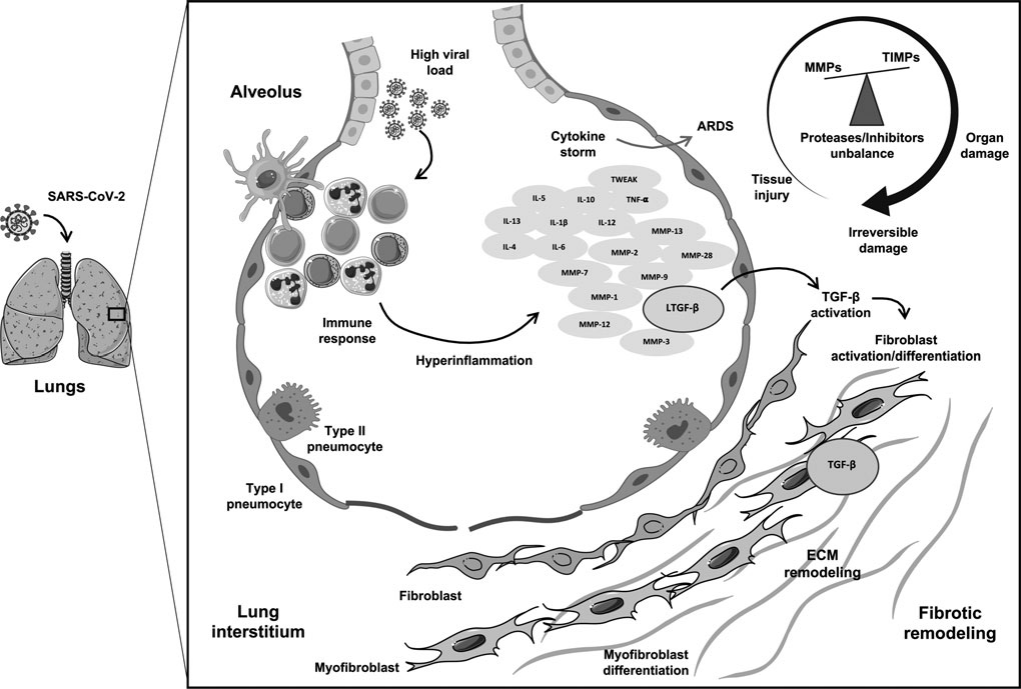
COVID-19 characteristic cytokine storm, highlighting the interplay between hyperinflammatory and tissue injury mediators, leading to pulmonary fibrosis. Schematic figure illustrating proposed mechanisms by which SARS-CoV-2 infection triggers a sustained dysregulated immune response in the lung resulting in a cytokine storm syndrome. ARDS may ensue by the upregulation of cytokines, chemokines, growth factors, and proteases such as metalloproteinases (MMPs) as well as their inhibitors (TIMPs), accompanied by a variety of complications according to disease severity. Growth factors, especially TGF-β, can be activated by cytokines and MMPs, promoting fibroblast proliferation and myofibroblast differentiation. These processes lead to altered ECM turnover, thus setting a profibrotic environment. ARDS, acute respiratory distress syndrome; COVID-19, coronavirus disease 2019; ECM, extracellular matrix; MMPs, matrix metalloproteinases; SARS-CoV-2, severe acute respiratory syndrome coronavirus-2; TGF-β, transforming growth factor beta; TIMPs, tissue inhibitors of metalloproteinases. The art pieces used in this figure were modified from Servier Medical Art by Servier and Biorender, licensed under a Creative Commons Attribution 3.0 Unported License.

Figure 1 depicts the severe COVID-19 characteristic cytokine storm and how hyperinflammatory and tissue injury mediators lead to PF.

## Postinfection PF: The Case of COVID-19

A remarkable feature of the convalescence phase of COVID-19 is that many patients who suffered severe lung damage remain with permanent pulmonary dysfunction. Many of the chronic deleterious effects of COVID-19 are related to post-injury PF. The mechanisms underlying the development of these complications are poorly defined so far. In patients with ARDS from other etiologies, severe epithelial and endothelial damage, accompanied by extensive fibrosis, is frequently observed. The patients who show the more remarkable fibrotic changes are those who required extended periods of mechanical ventilation (∼12 days) and developed more severe systemic organ failure (Ichikado and others 2012; Cabrera-Benitez and others 2014). Interestingly, PF is also a log-term sequela in pandemic influenza A (H1N1) patients.

Several mechanisms, including the barotrauma associated with mechanical ventilation, oxygen toxicity, and hyperinflammation, are crucial to determine the sequelae in these patients after recovering from severe disease (Xing and others 2015). These factors cause mild epithelial injuries that are not properly repaired, leading to fibroblast hyperactivation, excessive ECM deposition, and lung parenchyma remodeling (Yang and others 2020).

Cytokines also regulate tissue repair after injury besides providing the immune system with signals about the presence of external insults. However, to what extent mechanical injury and hyperinflammation contribute to the PF of COVID-19 is not understood. An important number of severe COVID-19 patients with ARDS require ICU admission and ventilator support, and those who survive show persistent ground-glass opacities, chronic pulmonary dysfunction, and PF that affect their quality of life (Ahmad Alhiyari and others 2020; George and others 2020; Xu and others 2020a). Meanwhile, hyperinflammation associated with severe COVID-19 also encompasses the release of inflammatory chemokines such as CCL2, CCL3, and IP-10.

These chemotactic factors are associated with a dysregulated activation of cells from the mononuclear phagocyte (MNP) compartment, promoting hyperinflammation in COVID-19 patients. Indeed, the bronchoalveolar fluid (BALF) of severe COVID-19 patients contains high concentrations of CCL2, CCL3, CCL4, and CCL7, and a decreased proportion of tissue-resident alveolar macrophages, but high amounts of inflammatory monocyte-derived macrophages (Liao and others 2020). Interestingly, these subpopulations of macrophages are enriched with transcripts that have been previously associated with tissue repair and promotion of fibrosis in liver cirrhosis (Ramachandran and others 2019; Merad and Martin 2020).

Remarkably, during fibrosis, many types of collagens can modulate the cellular functions and physiological processes of leukocytes and parenchymal cells. Also, in response to inflammation, the degradation of ECM by MMPs generates small peptides that can act as chemotactic factors for leukocytes, increasing the immunopathology of the disease. All these mechanisms further promote the hyperactivity of MMPs, thus causing progressive destruction of the lung parenchyma (Karsdal and others 2017). Hence, as discussed below, MMPs and other ECM components could act as readouts of ongoing profibrotic activity and lung injury in severe COVID-19 patients.

A critical controversy that has emerged recently is whether PF is an exclusive sequela of severe SARS-CoV-2 infection or patients with mild-to-moderate disease are also at risk. In this regard, evidence from both adult and pediatric convalescent COVID-19 patients suggests that older patients and those with more severe disease develop fibrosis (Antonio and others 2003; Ooi and others 2004; Chu and others 2006). Similarly, a study in Italy found that patients with nonsevere manifestations displaying pulmonary opacities showed complete remission of such lesions and no fibrosis during follow-up. In contrast, Dadhwal and Surani (2021) reported 5 cases of asymptomatic or mild symptomatic COVID-19 patients who presented shortness of breath and thorax images suggestive of resolving ground-glass opacities and later developed fibrosis 4–8 weeks after diagnosis (Rogliani and others 2020).

Together, these data highlight the necessity of more studies of convalescent COVID-19 patients to establish preventive and rehabilitation strategies against PF. For these purposes, novel biomarkers with predictive value for early detection of lung injury and fibrosis will also be required.

## MMPs in COVID-19: Inflammation, Lung Injury, and Fibrosis

Since SARS-CoV-2 infection triggers a characteristic cytokine storm, where molecules such as MMPs are overexpressed, their role in the pathogenesis of severe COVID-19 and SARS-CoV-2 infection sequela becomes of particular interest.

MMPs are a group of enzymes crucial for the homeostasis and turnover of several components of the ECM (Lagente and others 2005). They are a family of zinc-dependent endopeptidases belonging to the metzincin superfamily of proteinases, which mediate the cleavage of ECM components and other types of molecules such as receptors, growth factors, cytokines, and chemokines. MMPs can be classified into secreted-type and membrane-anchored. Also, these proteases can be categorized as collagenases, stromelysins, matrylysis, gelatinases, furin-activated, and other MMPs, depending on their substrate specificity and homology (Lagente and others 2005; Shiomi and others 2010; Karamanos and others 2021).

Under steady-state conditions, MMPs are poorly expressed in tissues. However, upon injury, inflammation, ECM turnover, and repair, their expression is enhanced. For instance, during ARDS, the expression of MMPs is dysregulated, and they might play a crucial role in the initiation and resolution of the disease (Albaiceta 2007). Indeed, ECM remodeling is a key pathogenic process in ARDS since morphometric analyses have shown ECM deposition and immune cell infiltration at acute and chronic stages of the disease.

Interestingly, severe COVID-19 patients who turn into ARDS exhibit disruption of the alveolar (epithelial)-capillary (endothelial) barrier in response to SARS-CoV-2 infection, intensifying fluid permeability and leukocyte extravasation (Matthay and others 2019; Hardy and Fernandez-Patron 2021). This leukocyte recruitment to the lungs in severe COVID-19 patients has not been yet fully characterized, but it might be regulated by specific leukocyte trafficking molecules, and if it is uncontrolled along with an enhanced proinflammatory response, it might lead to lung injury (Alon and others 2021).

During ARDS, both endothelial and epithelial injuries occur, and the late one can be produced directly by the virus, inflammatory cells, hyperoxia, hypoxia, among others, causing dissociation of intracellular junctions (Matthay and others 2019). In this scenario, MMPs increased levels in severe COVID-19 patients could be explained. It has been described that during the acute phases of ARDS, MMP-2 and MMP-9 mediate the repair of the alveolar epithelial–endothelial space injury induced by mechanical ventilation (Lagente and others 2005; González-López and others 2011), and also their expression is promoted by hypoxia and high-volume mechanical ventilation (Foda and others 2001; Liu and Khalil 2017). Interestingly, high MMP-2 concentrations correlate with a decrease of collagen levels and reestablishment of IL-10 expression in mice with ventilator-induced lung injury (González-López and others 2011).

Moreover, a study in BALF from ARDS patients with neutropenia and a model of neutropenic mice revealed a delay of lung injury repair. It also increases the levels of proinflammatory mediators such as TNF, IFN-γ, and macrophage inflammatory protein 2 (MIP-2) and the absence of MMP-9, strongly suggesting a crucial role of MMP-9 released from neutrophils for lung repair (Blázquez-Prieto and others 2018). Although MMP-2 and -9 are the most studied MMPs in ARDS, MMP-1, -3, -7, -8, -12, and -13 have also been found to increase in BALF from ARDS patients (O'Kane and others 2009; Davey and others 2011). Overexpression of MMP-1 and MMP-7 in serum is also a feature of patients with PF (Rosas and others 2008).

Another MMP upregulated during the initial phases of ARDS is MMP-28, and its transcript concentrations in BALF are associated with fewer ventilator-free days and increased alveolar neutrophils (Morrell and others 2020). Furthermore, it has been described in ARDS that neutrophil-derived mediators can also induce epithelial cell death by oxidation of soluble TNF ligand superfamily member 6 (FasL) and neutrophil extracellular traps (NETs) (Matthay and others 2019). Indeed, Pandolfi and others (2021) found higher concentrations of NETs in BALF of severe COVID-19 patients that correlated with neutrophil count, and also found in the lung tissue of COVID-19 deceased patients, epithelial and mesenchymal markers that evidence epithelial–mesenchymal transition (EMT).

Till date, scarce studies regarding the ECM remodeling process of COVID-19 have been performed. This is of major relevance, as we need a detailed understanding of the molecular components regulating cell migration, proliferation, and survival of fibroblasts and other parenchymal cells that mediate fibrosis to target these factors through novel antifibrotic COVID-19 treatments. Data from other human coronavirus infections show that these pathogens induce the expression of IL-6, TNF, and MCP-1 by modulating the activity of MMP-2 and MMP-9, which can be very helpful to understand SARS-CoV-2 pathogenesis. This induction of MMPs is further promoted by the same proinflammatory cytokines such as IL-6 and TNF and other soluble factors released upon viral infection (Giraudon and others 1997; Edwards and others 2000; Marten and Zhou 2005).

Recently, Shi and others (2020b) found higher serum concentrations of MMP-3 in COVID-19 patients than in healthy individuals, and their levels correlated with inflammatory markers such as IL-6 and IL-1β. However, when MMP-3 concentrations were longitudinally followed, they exhibited a decreasing trend. In another study in COVID-19 patients from a Norwegian cohort, MMP-9 was associated with respiratory failure and low PaO_2_/FiO_2_ (P/F) ratio values. In addition, longitudinal increases in MMP-9 from admission to 3–5 days after hospitalization were observed in those patients who developed respiratory failure (Ueland and others 2020). In a follow-up study of hospitalized patients with moderate and severe COVID-19, Safont and others (2022) reported that half of them exhibited impaired pulmonary diffusion, and the computed tomography (CT) scan from severe patients displayed fibrotic lesions and elevated serum PF biomarkers such as MMP1 and MMP7, both previously described as blood biomarkers in idiopathic PF (IPF) (Rosas and others 2008).

All this evidence points out to the induction of EMT not necessary by inflammation but rather by epithelial injury and the release of tissue injury markers, which could eventually lead to PF in severe COVID-19 patients who survive. This is in line with what Selman and others (2001) have previously described for IPF, where an aberrant activation of epithelial cells triggers the expression of mediators, such as MMPs and cytokines, that are involved in the migration, proliferation, and activation of fibroblasts, leading to their differentiation into myofibroblasts and the excessive and disorganized secretion of ECM (Selman and Pardo 2020). Therefore, these cellular mechanisms become of special interest for future research, especially to better understand the COVID-19 sequela, which has become a major health problem.

Interestingly, in a comparative study of critically ill COVID-19 and influenza A patients, among the immunological factors observed only in those with COVID-19 were increased concentrations of MMP-1 and -3, indicating a possible role of these MMPs in tissue damage associated with severe SARS-CoV-2 infection (Choreño-Parra and others 2021). As aforementioned, MMP-1 is linked to PF. This molecule is highly expressed in lung alveolar epithelial cells and activates IL-β, TNF, insulin growth factor-binding proteins, and several other cytokines (Rajah and others 1995; Hatfield and others 2010). Moreover, lung epithelial expression of MMP-1 correlates with reduced mitochondrial function, increase of HIF-1α expression, diminished production of reactive oxygen species (ROS), and promotion of a proliferative–migratory–antiapoptotic phenotype in alveolar epithelial cells (Herrera and others 2013).

On the contrary, MMP-3, also known as stromelysin-1, is broadly expressed in human tissues, cleaves several components of ECM, and activates other pro-MMPs such as pro-MMP-1, matrilysin, collagenases, and gelatinase B. Hence, MMP-3 is relevant to initiate the degradation process of ECM (Guizani and others 2021; Wan and others 2021). Also, MMP-3 is considered a proinflammatory factor inside several organs and linked to tissue damage, including nervous system inflammation and axonal degeneration, endothelial injury and inflammatory cell accumulation in the cardiovascular system, and cartilage matrix degradation with synovial hyperplasia in the musculoskeletal system (Wan and others 2021).

In addition, MMP-3 is increased in bronchial and alveolar epithelial cells, interstitial fibroblasts, alveolar macrophages, and other leukocytes in the lungs of patients with PF (Yamashita and others 2011; Craig and others 2015). In fact, MMP-3 has broad participation in fibrosing mechanisms, such as β-catenin signaling activation, latent TGF-β (LTGFB) activation, distal epithelial repair inhibition, and epithelial lung cell apoptosis induction (Craig and others 2015).

MMPs have long been considered proteases implicated in degrading matrix proteins. Nevertheless, they also have critical roles in the resolution of acute inflammation. In murine models and patients with acute inflammation, macrophage production of MMP-10, -19, and -28 may have a beneficial effect against excessive inflammation (Fingleton 2017). Another relevant macrophage-derived MMP is MMP-12, which has anti-inflammatory protective roles. Proteomic analyses have identified direct substrates of MMP-12 during inflammation. These include acute-phase proteins, coagulation proteins, and complement proteins. Indeed, MMP-12 inactivates C3, C3a, and C5a, thus reducing complement system inflammatory and chemotactic activities. Interestingly, this complement inactivation also promoted macrophages' phagocytic activity, facilitating inflammation resolution, reducing lung injury, and decreasing neutrophil influx into the alveolar spaces, as observed in MMP-12-deficient murine models (Warner and others 2001; Bellac and others 2014).

Furthermore, it has been described that the minor allele of a single nucleotide polymorphism in MMP-12, rs2276109, is associated with a reduced risk for developing cystic fibrosis and chronic obstructive pulmonary disease (Hunninghake and others 2009; Trojanek and others 2014). Together, these data suggest that MMP-12 could be a key mediator in the resolution of hyperinflammation in COVID-19 patients. Hence, future studies should evaluate MMP-12 as an anti-inflammatory mediator that could promote the resolution of the disease.

Finally, turnover of ECM components by MMPs might be influenced directly by SARS-CoV-2 and alter some aspects of the host–pathogen interactions that are important to determine the severity of the disease. As such, binding of the S protein to ACE2 affects the regulatory activity of angiotensin II, increasing inflammation and MMP activation and leading to further disruption of ECM (Guizani and others 2021). Proof of this concept comes from studies showing that cells infected with SARS-CoV-2 lack major components of ECM, some of them essential to maintain ECM homeostasis, such as ANXA2 (Anzueto 2002). Also, during SARS-CoV infection, tissue inhibitors of metalloproteinases (TIMPs) are secreted possibly to maintain ECM equilibrium. Particularly, TIMP-2 inhibits a disintegrin and metalloprotease 17 (ADAM-17), which is responsible for shedding the ACE2 receptor, hence implicated in the propagation of the infection.

In summary, the relevant role of MMPs in pulmonary injury and lung repair processes as regulators of other molecules such as cytokines, chemokines, and growth factors should be considered to better understand the pathogenesis and outcomes of severe COVID-19.

## TGF-β in COVID-19 Immunity and PF

Just as MMPs, TGF-β could have a crucial role during respiratory viral infections by mediating both suppression of innate immune responses and lung ECM remodeling. The TGFB cytokine family is composed of 3 dimeric polypeptide growth factor isoforms, TGF-β1, TGF-β2, and TGF-β3, which have crucial roles in cell differentiation, proliferation, and migration. These growth factors also have relevant functions in processes involved in developing diseases such as fibrosis, wound healing, carcinogenesis, and immune response regulation (Li and others 2006; Thomas and others 2016). TGF-β is synthesized as a precursor protein, the LTGFB, which must be activated by cleaving its C-terminal- and N-terminal-associated peptides. This activation occurs by a different mechanism such as proteolysis, low pH, thrombospondin-1, ROS, and the action of integrins upon traction forces or stiffness of the ECM during disease (Crawford and others 1998; Munger and others 1999; Annes and others 2003; Shi and others 2011).

The TGF-β signaling pathway starts once it binds to a cell through the homodimer of TGF-β type II receptors (TβRII), which along with the homodimer of TGF-β type I receptor (TβRI) contributes to the formation of a stable complex. Then, TβRII autophosphorylates and catalyzes the transphosphorylation of TβRI, activating its kinase activity and phosphorylating in turn the small mothers against decapentaplegic (SMAD) proteins (Ong and others 2021).

Besides all the abovementioned activities, TGF-β also plays significant functions in innate and adaptive immune responses. In fact, most immune cell populations can produce and secrete TGF-β during defense against infection, although its overproduction inhibits adequate immune responses. This has been observed in several respiratory viral infections with rhinovirus (RV), respiratory syncytial virus (RSV), human metapneumovirus, and influenza A and B viruses (Thomas and others 2016). These pathogens may evade immune responses by directly regulating TGF-β production and activation. Interestingly, some viral proteins can trigger the production of TGF-β, elucidating thus that the pathogen–host interactions may influence TGF-β activity. For instance, it has been reported that the neuraminidase (NA) of the influenza A virus directly activates LTGFB by removing sialic acid motifs from its surface (Schultz-Cherry and Hinshaw 1996).

The role of TGF-β during SARS-CoV-2 infection is not clear at the moment. The activation of LTGFB has been reported to be induced by the papain-like protease (PL-pro) from SARS-CoV (Li and others 2016). This viral deubiquitinating enzyme inactivates type I IFN signaling pathways at several levels and potentiates the expression of TGF-β1. Nonetheless, there are no reports of any SARS-CoV-2 protease that can activate LTGFB. Of note, when comparing asymptomatic versus symptomatic COVID-19 patients, Montalvo-Villalba and colleagues found that in the early inflammatory phase of the immune response against the virus, symptomatic patients presented lower transcript levels of TGF-β1 and the regulated upon activation, normal T cell expressed and presumably secreted (RANTES) protein in the upper airway. They also found a significant negative correlation between IFN-γ and TGF-β1 in asymptomatic patients, suggesting that TGF-β1 could regulate IFN-γ expression in these individuals (Montalvo Villalba and others 2020).

This finding coincides with previous ones demonstrating that epithelial-derived TGF-β acts as a proviral factor suppressing early immune responses during influenza A virus infection. In addition, mice specifically lacking bronchial epithelial TGF-β1 expression exhibited impaired viral replication, all of these results suggesting that epithelial-derived TGF-β1 suppresses early IFN-γ responses, which favors an increased viral burden and lung pathology (Denney and others 2018). All these data may indicate that, during viral lung infections, early epithelial TGF-β secretion exerts a local immune regulation that is detrimental to the host. Hence, TGF-β could have a certain prognostic value as a biomarker for COVID-19 that deserves further investigation.

TGF-β might also participate in the mechanisms of ECM remodeling underlying post-COVID-19 sequelae, as it was revealed by Colarusso and others (2021) who found higher TGF-β, CXCL10, and IL-1α plasma levels in post-COVID-19 patients who exhibited ground-glass opacities in the chest CT scan. Indeed, in patients with PF, TGF-β1 is involved in myofibroblast differentiation from fibroblasts, and α-smooth muscle actin (α-SMA)-expressing differentiated myofibroblasts mediate the production of ECM components, integrins, protease inhibitors, regulators of small GTPases, and MMPs. TGF-β1 also inhibits the production of antifibrotic molecules such as prostaglandin E2 and represses epithelial cell growth and repair (Garrison and others 2013; Saito and others 2018). Furthermore, TGF-β interacts with other molecules that have important functions for ECM turnover. In this regard, it should be noted that in tumor cells, TGF-β is activated by the proteolytic activity of MMPs, such as MMP-2, -9, -13, and -14.

Interestingly, a TGF-β-positive regulatory loop has also been described, since TGF-β also induces the expression of these proteases, contributing to potentiate tumor progression (Quintanilla and others 2012). Whether the same positive loop occurs during viral infections such as COVID-19 contributing to alterations in ECM remodeling remains to be elucidated. Some studies in severe COVID-19 patients have revealed that an early TGF-β production is associated with the impairment of immune cells such as NK cells and B cells (Ferreira-Gomes and others 2021; Witkowski and others 2021), which contributes to a failure of the host early viral clearance. This TGF-β production becomes relevant since it is a hallmark of uninterrupted Th17 cell activation (Lee and others 2012). In fact, Sadeghi and others (2021) reported that severe COVID-19 patients have an enhanced Th17 cell response and a decreased Treg cell response, suggesting that this imbalanced Th17/Treg cell ratio is critical in the disease pathogenesis.

**FIG. 2.**
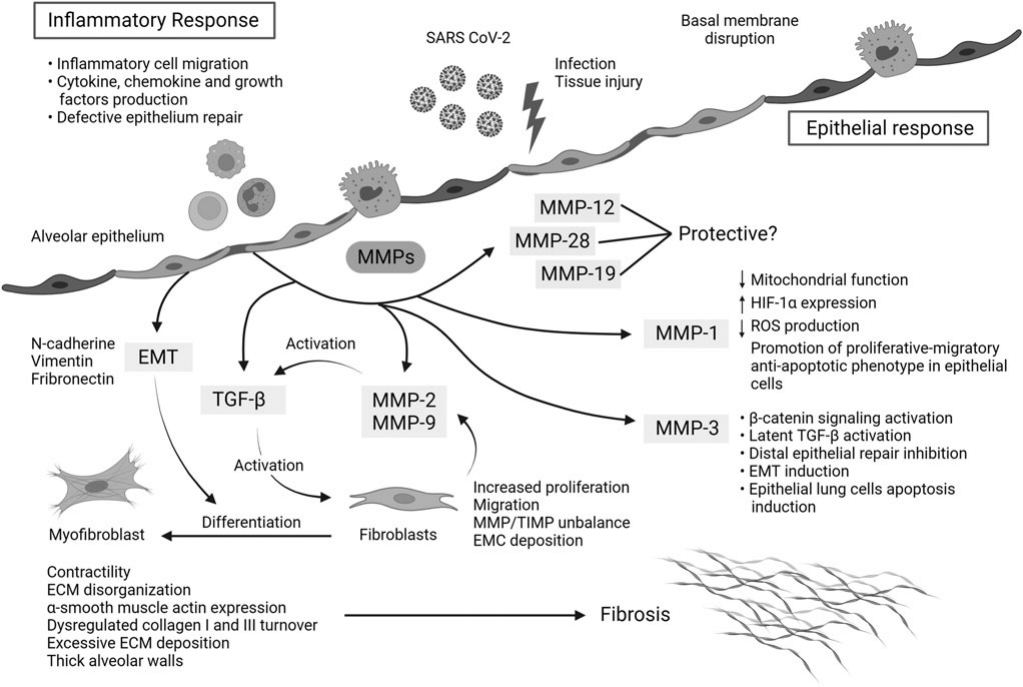
Mechanisms of action of MMPs and TGF-β that link hyperinflammation and pulmonary fibrosis in severe COVID-19. The replication rate of SARS-CoV-2 and associated lung epithelial cell death may trigger a cytokine storm, hyperinflammation, and epithelial dysfunction. During severe COVID-19, the initial acute inflammatory response might be followed by epithelial cell hyperplasia, where key mediators such as MMPs drive epithelial and endothelial injury and uncontrolled fibroblast proliferation. Moreover, some MMPs can activate other profibrotic mediators such as TGF-β, which also contributes to fibroblast accumulation, differentiation to myofibroblasts, ECM disorganization, dysregulated collagen I and III turnover, excessive ECM deposition, and thick alveolar walls. MMPs can also be involved in the regulation of other pathways, such as MMP-1, implicated in the reduction of mitochondrial function, the reduction of reactive oxygen species, and the overexpression of HIF-1α, and MMP3 in latent TGF-β activation, β-catenin signaling activation, and lung epithelial cell apoptosis, among others. The art pieces used in this figure were modified from Servier Medical Art by Servier and Biorender, licensed under a Creative Commons Attribution 3.0 Unported License.

Figure 2 summarizes possible mechanisms by which MMPs and TGF-β are involved in severe COVID-19 and link with PF sequela.

## Potential Therapeutics for Severe COVID-19 Targeting ECM Remodeling

The impact of COVID-19 requires a massive retaliation with diagnostic and therapeutic innovations that, along with vaccines, could reduce disease morbidity and mortality. Importantly, the real implications of this global pandemic are not completely visible yet, as it is predicted that sequela will affect global public health, especially among those recovered from severe disease. Hence, innovative strategies against COVID-19 should focus on limiting chronic complications too. Unfortunately, therapeutic options for COVID-19 have not had the same positive outcomes as vaccines so far.

Hence, as discussed through the article, we should consider lessons from previous respiratory virus outbreaks. Clinical trials will also be crucial to compare asymptomatic, mild, moderate, severe, and convalescent COVID-19 patients.

Current approaches for host-directed therapy against SARS-CoV-2 rely on the blockade of proinflammatory mediators, such as IL-6 with tocilizumab. Although this treatment might reduce inflammation, it does not have a significant clinical benefit for patients with COVID-19 (Alattar and others 2020; Lan and others 2020). There is also little evidence about the effects of tocilizumab on post-COVID-19 sequela. A combination therapy directed to anti-inflammatory and antifibrotic pathways with pirfenidone and IL-1 or IL-6 inhibitors has been proposed (Ferrara and others 2020), but further evidence is required to make reliable conclusions.

Other antifibrotic therapies for COVID-19 under investigation in clinical trials have pleiotropic effects and are mainly used for chronic disease management rather than as preventive treatments. Among these are pirfenidone and nintedanib, which effectively mitigate the rate of lung function decline in PF patients, have anti-inflammatory properties, and do not exhibit immunosuppressive effects (George and others 2020). Pirfenidone is an oral antifibrotic and anti-inflammatory agent that reduces lung function decline, decreases mortality, and improves progression-free survival in PF patients. This agent also reduces serum and lung IL-6 concentrations making it a good candidate for COVID-19 treatment (Lancaster and others 2017; Crisan-Dabija and others 2020). Nintedanib is a tyrosine kinase inhibitor, which suppresses processes involved in the progression of PF (Flaherty and others 2019).

Interestingly, Rao and others (2020) performed a proteomic-wide Mendelian randomization study, in which 2 proteins inhibited by nintedanib were linked to elevated ACE2, suggesting that this antifibrotic could lower ACE2 expression, thus also reducing susceptibility to SARS-CoV-2 infection (Crisan-Dabija and others 2020).

Emerging evidence summarized here suggests interdependent roles of hyperinflammation and impaired lung tissue healing in severe COVID-19 and its sequela, emphasizing the potential of several MMPs and TGF-β as biomarkers and therapeutic targets. In-depth analysis of certain specific MMPs could provide further insights into the pathobiology of COVID-19 and open new avenues for diagnostic and treatment development. For instance, MMP-3 and -9 could serve as prognostic markers of severity and risk of PF (Shi and others 2020b; Ueland and others 2020). MMP-1 and -3 may be helpful to differentiate COVID-19 from pandemic influenza A (H1N1) and predict the risk of severe manifestations as well. Targeting these molecules might also have a particular therapeutic value that deserves further investigation.

We also presented evidence that suggests that TGF-β plays an important role during viral infections impeding protective immunity and the resolution of disease. Hence, blockade of TGF-β function with antibodies, inhibitors, or by activating the bone morphogenic protein (BMP) signaling pathway with agonists to reverse the effects might reduce morbidity and long-term sequela (Carlson and others 2020; Chen 2020; Ong and others 2021). Additional antifibrotic agents targeting various molecules involved in the fibrosing pathway downstream of TGF-β, including avβ6 integrin (BG00011), galectins (TD139), rapamycin, JNK inhibitor (PRM-151), the agonist of ACE2 receptor (C21), and phosphodiesterase type 5 inhibitors PDE5-i (Crisan-Dabija and others 2020), also warrant more interest.

## Conclusions

In conclusion, the interaction between cytokines and molecules mediating ECM remodeling, such as MMPs and TGF-β, might significantly impact the pathogenesis of respiratory viral infections.

In this regard, new evidence reveals a more complex interplay among inflammatory and tissue injury mediators that have influence in the disease severity and in its sequela. Severe COVID-19 characteristic cytokine storm is crucial into understanding the disease outcome. Also, the time and tissue specificity of the expression of key mediators, such as MMPs and TGF-β, could define the clinical outcome of the patient, and also be useful as diagnostic and prognostic markers.

Even if some studies are shedding light into the possible action mechanisms of MMPs, there is still a lot to find out about the role of these mediators into the development of severe COVID-19 and its sequela. Lessons learned from other pathologies such as other coronavirus infections, influenza A virus infection or IPF, could be helpful into getting answers about the complex mechanisms that occur during SARS-CoV-2 infection, and could provide valuable knowledge for the founding of diagnostic and prognostic tools for severe COVID-19 and its sequela, which is now a worldwide major health problem. Finally, targeting these molecules might also have a particular therapeutic value, such as TGF-β blockade with antibodies, inhibitors, or agonists, to reverse its effects that could reduce morbidity and prevent long-term sequela such as PF development.

More interest in these factors might provide essential insights to understand better the processes behind poor clinical outcomes and sequela of severe COVID-19 and novel targets for translational applications in pulmonary medicine.

## References

[B1] Abbas AK, Trotta E, Simeonov DR, Marson A, Bluestone JA. 2018. Revisiting IL-2: biology and therapeutic prospects. Sci Immunol 3(25):eaat1482. 29980618 10.1126/sciimmunol.aat1482

[B2] Ahmad Alhiyari M, Ata F, Islam Alghizzawi M, Bint I Bilal A, Salih Abdulhadi A, Yousaf Z. 2020. Post COVID-19 fibrosis, an emerging complicationof SARS-CoV-2 infection. IDCases 23:e01041. 33425682 10.1016/j.idcr.2020.e01041 PMC7785952

[B3] Alattar R, Ibrahim TBH, Shaar SH, Abdalla S, Shukri K, Daghfal JN, Khatib MY, Aboukamar M, Abukhattab M, Alsoub HA, Almaslamani MA, Omrani AS. 2020. Tocilizumab for the treatment of severe coronavirus disease 2019. J Med Virol 92(10):2042–2049. 32369191 10.1002/jmv.25964 PMC7267594

[B4] Albaiceta GM, Fueyo A. 2007. Matrix metalloproteinases in acute lung injury. Berlin, Heidelberg: Springer.

[B5] Alon R, Sportiello M, Kozlovski S, Kumar A, Reilly EC, Zarbock A, Garbi N, Topham DJ. 2021. Leukocyte trafficking to the lungs and beyond: lessons from influenza for COVID-19. Nat Rev Immunol 21(1):49–64. 33214719 10.1038/s41577-020-00470-2 PMC7675406

[B6] Annes JP, Munger JS, Rifkin DB. 2003. Making sense of latent TGFbeta activation. J Cell Sci 116(Pt 2):217–224. 12482908 10.1242/jcs.00229

[B7] Antonio GE, Wong KT, Hui DS, Wu A, Lee N, Yuen EH, Leung CB, Rainer TH, Cameron P, Chung SS, Sung JJ, Ahuja AT. 2003. Thin-section CT in patients with severe acute respiratory syndrome following hospital discharge: preliminary experience. Radiology 228(3):810–815. 12805557 10.1148/radiol.2283030726

[B8] Anzueto A. 2002. Exogenous surfactant in acute respiratory distress syndrome: more is better. Eur Respir J 19(5):787–789. 12030713 10.1183/09031936.02.00284902

[B9] Bardhan K, Anagnostou T, Boussiotis VA. 2016. The PD1:PD-L1/2 pathway from discovery to clinical implementation. Front Immunol 7:550. 28018338 10.3389/fimmu.2016.00550 PMC5149523

[B10] Becker R. 2020. COVID-19 update: Covid-19-associated coagulopathy. J Thromb Thrombolysis 50(1):54–67. 32415579 10.1007/s11239-020-02134-3 PMC7225095

[B11] Beckert H, Meyer-Martin H, Buhl R, Taube C, Reuter S. 2020. Single and synergistic effects of type 2 cytokines on eosinophils and asthma hallmarks. J Immunol 204(3):550–558. 31862712 10.4049/jimmunol.1901116

[B12] Bekele Y, Sui Y, Berzofsky JA. 2021. IL-7 in SARS-CoV-2 infection and as a potential vaccine adjuvant. Front Immunol 12:737406. 34603318 10.3389/fimmu.2021.737406 PMC8484798

[B13] Bellac CL, Dufour A, Krisinger MJ, Loonchanta A, Starr AE, Auf dem Keller U, Lange PF, Goebeler V, Kappelhoff R, Butler GS, Burtnick LD, Conway EM, Roberts CR, Overall CM. 2014. Macrophage matrix metalloproteinase-12 dampens inflammation and neutrophil influx in arthritis. Cell Rep 9(2):618–632. 25310974 10.1016/j.celrep.2014.09.006

[B14] Bernard GR, Artigas A, Brigham KL, Carlet J, Falke K, Hudson L, Lamy M, Legall JR, Morris A, Spragg R. 1994. The American-European Consensus Conference on ARDS. Definitions, mechanisms, relevant outcomes, and clinical trial coordination. Am J Respir Crit Care Med 149(3 Pt 1):818–824. 10.1164/ajrccm.149.3.7509706 7509706

[B15] Blázquez-Prieto J, López-Alonso I, Amado-Rodríguez L, Huidobro C, González- López A, Kuebler WM, Albaiceta GM. 2018. Impaired lung repair during neutropenia can be reverted by matrix metalloproteinase-9. Thorax 73(4):321–330. 28947666 10.1136/thoraxjnl-2017-210105

[B16] Bonaventura A, Vecchié A, Wang TS, Lee E, Cremer PC, Carey B, Rajendram P, Hudock KM, Korbee L, Van Tassell BW, Dagna L, Abbate A. 2020. Targeting GM-CSF in COVID-19 pneumonia: rationale and strategies. Front Immunol 3(11):1625. 10.3389/fimmu.2020.01625 PMC7348297 32719685

[B17] Bouezzedine F, Fardel O, Gripon P. 2015. Interleukin 6 inhibits HBV entry through NTCP down regulation. Virology 481:34–42. 25765005 10.1016/j.virol.2015.02.026

[B18] Buszko M, Nita-Lazar A, Park JH, Schwartzberg PL, Verthelyi D, Young HA, Rosenberg AS. 2021. Lessons learned: new insights on the role of cytokines in COVID-19. Nat Immunol 22(4):404–411. 33723418 10.1038/s41590-021-00901-9 PMC9317654

[B19] Cabrera-Benitez NE, Laffey JG, Parotto M, Spieth PM, Villar J, Zhang H, Slutsky AS. 2014. Mechanical ventilation-associated lung fibrosis in acute respiratory distress syndrome: a significant contributor to poor outcome. Anesthesiology 121(1):189–198. 24732023 10.1097/ALN.0000000000000264 PMC4991945

[B20] Cao X. 2020. COVID-19: immunopathology and its implications for therapy. Nat Rev Immunol 20(5):269–270. 32273594 10.1038/s41577-020-0308-3 PMC7143200

[B21] Carlson FR Jr, Bosukonda D, Keck PC, Carlson WD. 2020. Multiorgan damage in patients with COVID-19: is the TGFB/BMP pathway the missing link? JACC Basic Transl Sci 5(11):1145–1148. 32984657 10.1016/j.jacbts.2020.09.003 PMC7508496

[B22] Chen W. 2020. A potential treatment of COVID-19 with TGF-β blockade. Int J Biol Sci 16(11):1954–1955. 32398962 10.7150/ijbs.46891 PMC7211163

[B23] Choreño-Parra JA, Jiménez-Álvarez LA, Cruz-Lagunas A, Rodríguez-Reyna TS, Ramírez-Martínez G, Sandoval-Vega M, Hernández-García DL, Choreño-Parra EM, Balderas-Martínez YI, Martinez-Sánchez ME, Márquez-García E, Sciutto E, Moreno-Rodríguez J, Barreto-Rodríguez JO, Vázquez-Rojas H, Centeno-Sáenz GI, Alvarado-Peña N, Salinas-Lara C, Sánchez-Garibay C, Galeana-Cadena D, Hernández G, Mendoza-Milla C, Domínguez A, Granados J, Mena-Hernández L, Pérez-Buenfil L, Domínguez-Cheritt G, Cabello-Gutiérrez C, Luna-Rivero C, Salas-Hernández J, Santillán-Doherty P, Regalado J, Hernández-Martínez A, Orozco L, Ávila-Moreno F, García-Latorre EA, Hernández-Cárdenas CM, Khader SA, Zlotnik A, Zúñiga J. 2021. Clinical and immunological factors that distinguish COVID-19 from pandemic influenza A (H1N1). Front Immunol 12:593595. 33995342 10.3389/fimmu.2021.593595 PMC8115405

[B24] Chu WC LA, Ng AW, So HK, Lam WW, Lo KL, Yeung MC, Yau YS, Chiu WK, Leung CW, Ng PC, Hon KL, Mo KW, Fok TF, Ahuja AT. 2006. Thin-section CT 12 months after the diagnosis of severe acute respiratory syndrome in pediatric patients. AJR Am J Roentgenol 186(6):1707–1714. 16714663 10.2214/AJR.05.0382

[B25] Colarusso C, Maglio A, Terlizzi M, Vitale C, Molino A, Pinto A, Vatrella A, Sorrentino R. 2021. Post-COVID-19 patients who develop lung fibrotic-like changes have lower circulating levels of IFN-β but higher levels of IL-1α and TGF-β. Biomedicines 9(12):1931. 34944747 10.3390/biomedicines9121931 PMC8698335

[B26] Costela-Ruiz VJ, Illescas-Montes R, Puerta-Puerta JM, Ruiz C, Melguizo-Rodríguez L. 2020. SARS-CoV-2 infection: the role of cytokines in COVID-19 disease. Cytokine Growth Factor Rev 54:62–75. 32513566 10.1016/j.cytogfr.2020.06.001 PMC7265853

[B27] Craig VJ, Zhang L, Hagood JS, Owen CA. 2015. Matrix metalloproteinases as therapeutic targets for idiopathic pulmonary fibrosis. Am J Respir Cell Mol Biol 53(5):585–600. 26121236 10.1165/rcmb.2015-0020TR PMC4742954

[B28] Crawford SE, Stellmach V, Murphy-Ullrich JE, Ribeiro SM, Lawler J, Hynes RO, Boivin GP, Bouck N. 1998. Thrombospondin-1 is a major activator of TGF-beta1 in vivo. Cell 93(7):1159–1170. 9657149 10.1016/s0092-8674(00)81460-9

[B29] Crisan-Dabija R, Pavel CA, Popa IV, Tarus A, Burlacu A. 2020. “A chain only as strong as its weakest link”: an up-to-date literature review on the bidirectional interaction of pulmonary fibrosis and COVID-19. J Proteome Res 19(11):4327–4338. 32883081 10.1021/acs.jproteome.0c00387

[B30] Dadhwal R, Sharma M, Surani S. 2021. Restrictive lung disease in patients with subclinical coronavirus infection: are we bracing ourselves for devastating sequelae? Cureus 13(1):e12501. 33564509 10.7759/cureus.12501 PMC7861059

[B31] Davey A, McAuley DF, O'Kane CM. 2011. Matrix metalloproteinases in acute lung injury: mediators of injury and drivers of repair. Eur Respir J 38(4):959–970. 21565917 10.1183/09031936.00032111

[B32] de Bree GJ, van Leeuwen EM, Out TA, Jansen HM, Jonkers RE, van Lier RA. 2005. Selective accumulation of differentiated CD8^+^ T cells specific for respiratory viruses in the human lung. J Exp Med 202(10):1433–1442. 16301748 10.1084/jem.20051365 PMC2212987

[B33] de Wit E, van Doremalen N, Falzarano D, Munster VJ. 2016. SARS and MERS: recent insights into emerging coronaviruses. Nat Rev Microbiol 14(8):523–534. 27344959 10.1038/nrmicro.2016.81 PMC7097822

[B34] Denney L, Branchett W, Gregory LG, Oliver RA, Lloyd CM. 2018. Epithelial-derived TGF-β1 acts as a pro-viral factor in the lung during influenza A infection. Mucosal Immunol 11(2):523–535. 29067998 10.1038/mi.2017.77 PMC5797694

[B35] Diao B, Wang C, Tan Y, Chen X, Liu Y, Ning L, Chen L, Li M, Liu Y, Wang G, Yuan Z, Feng Z, Zhang Y, Wu Y, Chen Y. 2019. Reduction and functional exhaustion of T cells in patients with coronavirus disease 2019 (COVID-19). Front Immunol 11:827. 10.3389/fimmu.2020.00827 PMC7205903 32425950

[B36] Donlan AN, Sutherland TE, Marie C, Preissner S, Bradley BT, Carpenter RM, Sturek JM, Ma JZ, Moreau GB, Donowitz JR, Buck GA, Serrano MG, Burgess SL, Abhyankar MM, Mura C, Bourne PE, Preissner R, Young MK, Lyons GR, Loomba JJ, Ratcliffe SJ, Poulter MD, Mathers AJ, Day AJ, Mann BJ, Allen JE, Petri WA Jr. 2021. IL-13 is a driver of COVID-19 severity. JCI Insight 6(15):e150107. 10.1172/jci.insight.150107 PMC8410056 34185704

[B37] Du F, Liu B, Zhang S. 2021. COVID-19: the role of excessive cytokine release and potential ACE2 down-regulation in promoting hypercoagulable state associated with severe illness. J Thromb Thrombolysis 51(2):313–329. 32676883 10.1007/s11239-020-02224-2 PMC7365308

[B38] Edwards JA, Denis F, Talbot PJ. 2000. Activation of glial cells by human coronavirus OC43 infection. J Neuroimmunol 108(1–2):73–81. 10900340 10.1016/S0165-5728(00)00266-6 PMC7119868

[B39] Fathi N, Rezaei N. 2020. Lymphopenia in COVID-19: therapeutic opportunities. Cell Biol Int 44(9):1792–1797. 32458561 10.1002/cbin.11403 PMC7283672

[B40] Ferrara F, Granata G, Pelliccia C, La Porta R, Vitiello A. 2020. The added value of pirfenidone to fight inflammation and fibrotic state induced by SARS-CoV-2: anti-inflammatory and anti-fibrotic therapy could solve the lung complications of the infection? Eur J Clin Pharmacol 76(11):1615–1618. 32594204 10.1007/s00228-020-02947-4 PMC7320911

[B41] Ferreira-Gomes M, Kruglov A, Durek P, Heinrich F, Tizian C, Heinz GA, Pascual-Reguant A, Du W, Mothes R, Fan C, Frischbutter S, Habenicht K, Budzinski L, Ninnemann J, Jani PK, Guerra GM, Lehmann K, Matz M, Ostendorf L, Heiberger L, Chang HD, Bauherr S, Maurer M, Schönrich G, Raftery M, Kallinich T, Mall MA, Angermair S, Treskatsch S, Dörner T, Corman VM, Diefenbach A, Volk HD, Elezkurtaj S, Winkler TH, Dong J, Hauser AE, Radbruch H, Witkowski M, Melchers F, Radbruch A, Mashreghi MF. 2021. ARS-CoV-2 in severe COVID-19 induces a TGF-β-dominated chronic immune response that does not target itself. Nat Commun 12(1):1961. 33785765 10.1038/s41467-021-22210-3 PMC8010106

[B42] Fingleton B. 2017. Matrix metalloproteinases as regulators of inflammatory processes. Biochim Biophys Acta Mol Cell Res 1864(11 Pt A):2036–2042. 28502592 10.1016/j.bbamcr.2017.05.010

[B43] Flaherty KR, Wells AU, Cottin V, Devaraj A, Walsh SLF, Inoue Y, Richeldi L, Kolb M, Tetzlaff K, Stowasser S, Coeck C, Clerisme-Beaty E, Rosenstock B, Quaresma M, Haeufel T, Goeldner RG, Schlenker-Herceg R, Brown KK; INBUILD Trial Investigators. 2019. Nintedanib in progressive fibrosing interstitial lung diseases. N Engl J Med 381(18):1718–1727. 31566307 10.1056/NEJMoa1908681

[B44] Foda HD, Rollo EE, Drews M, Conner C, Appelt K, Shalinsky DR, Zucker S. 2001. Ventilator-induced lung injury upregulates and activates gelatinases and EMMPRIN: attenuation by the synthetic matrix metalloproteinase inhibitor, Prinomastat (AG3340). Am J Respir Cell Mol Biol 25(6):717–724. 11726397 10.1165/ajrcmb.25.6.4558f

[B45] Garrison G, Huang SK, Okunishi K, Scott JP, Kumar Penke LR, Scruggs AM, Peters-Golden M. 2013. Reversal of myofibroblast differentiation by prostaglandin E(2). Am J Respir Cell Mol Biol 48(5):550–558. 23470625 10.1165/rcmb.2012-0262OC PMC3707380

[B46] George PM, Wells AU, Jenkins RG. 2020. Pulmonary fibrosis and COVID-19: the potential role for antifibrotic therapy. Lancet Respir Med 8:807–815. 32422178 10.1016/S2213-2600(20)30225-3 PMC7228727

[B47] Giraudon P, Buart S, Bernard A, Belin MF. 1997. Cytokines secreted by glial cells infected with HTLV-I modulate the expression of matrix metalloproteinases (MMPs) and their natural inhibitor (TIMPs): possible involvement in neurodegenerative processes. Mol Psychiatry 2(2):107–110. 9106228 10.1038/sj.mp.4000218

[B48] González-López A, Astudillo A, García-Prieto E, Fernández-García MS, López-Vázquez A, Batalla-Solís E, Taboada F, Fueyo A, Albaiceta GM. 2011. Inflammation and matrix remodeling during repair of ventilator-induced lung injury. Am J Physiol Lung Cell Mol Physiol 301(4):L500–L509. 21743031 10.1152/ajplung.00010.2011

[B49] Gorski SA, Lawrence MG, Hinkelman A, Spano MM, Steinke JW, Borish L, Teague WG, Braciale TJ. 2019. Expression of IL-5 receptor alpha by murine and human lung neutrophils. PLoS One 14(8):e0221113. 31415658 10.1371/journal.pone.0221113 PMC6695150

[B50] Grasselli G, Zangrillo A, Zanella A, Antonelli M, Cabrini L, Castelli A, Cereda D, Coluccello A, Foti G, Fumagalli R, Iotti G, Latronico N, Lorini L, Merler S, Natalini G, Piatti A, Ranieri MV, Scandroglio AM, Storti E, Cecconi M, Pesenti A; COVID-19 Lombardy ICU Network. 2020. Baseline characteristics and outcomes of 1591 patients infected with SARS-CoV-2 admitted to ICUs of the Lombardy region, Italy. JAMA 323(16):1574–1581. 32250385 10.1001/jama.2020.5394 PMC7136855

[B51] Guan WJ, Ni Z, Hu Y, Liang WH, Ou CQ, He JX, Liu L, Shan H, Lei CL, Hui DSC, Du B, Li L, Zeng G, Yuen KY, Chen RC, Tang CL, Wang T, Chen PY, Xiang J, Li SY, Wang JL LZ, Peng YX, Wei L, Liu Y, Hu YH, Peng P, Wang JM, Liu JY, Chen, Z LG, Zheng ZJ, Qiu SQ, Luo J, Ye CJ, Zhu SY, Zhong NS; China Medical Treatment Expert Group for Covid-19. 2020. Clinical characteristics of coronavirus disease 2019 in China. N Engl J Med 382(28):1708–1720. 32109013 10.1056/NEJMoa2002032 PMC7092819

[B52] Gubernatorova EO, Namakanova OA, Gorshkova EA, Medvedovskaya AD, Nedospasov SA, Drutskaya MS. 2021. Novel anti-cytokine strategies for prevention and treatment of respiratory allergic diseases. Front Immunol 12:601842. 34084159 10.3389/fimmu.2021.601842 PMC8167041

[B53] Guillamat-Prats R, Camprubí-Rimblas M, Bringué J, Tantinyà N, Artigas A. 2017. Cell therapy for the treatment of sepsis and acute respiratory distress syndrome. Ann Transl Med 5(22):446. 29264363 10.21037/atm.2017.08.28 PMC5721220

[B54] Guizani I, Fourti N, Zidi W, Feki M, Allal-Elasmi M. 2021. SARS-CoV-2 and pathological matrix remodeling mediators. Inflamm Res 70(8):847–858. 34286362 10.1007/s00011-021-01487-6 PMC8294315

[B55] Hardy E, Fernandez-Patron C. 2021. Targeting MMP-regulation of inflammation to increase metabolic tolerance to COVID-19 pathologies: a hypothesis. Biomolecules 11(3):390. 33800947 10.3390/biom11030390 PMC7998259

[B56] Harker JA, Lewis GM, Mack L, Zuniga EI. 2011. Late interleukin-6 escalates T follicular helper cell responses and controls a chronic viral infection. Science 334(6057):825–829. 21960530 10.1126/science.1208421 PMC3388900

[B57] Hatfield KJ, Reikvam H, Bruserud Ø. 2010. The crosstalk between the matrix metalloprotease system and the chemokine network in acute myeloid leukemia. Curr Med Chem 17(36):4448–4461. 21062258 10.2174/092986710794183033

[B58] Herrera I, Cisneros J, Maldonado M, Ramírez R, Ortiz-Quintero B, Anso E, Chandel NS, Selman M, Pardo A. 2013. Matrix metalloproteinase (MMP)-1 induces lung alveolar epithelial cell migration and proliferation, protects from apoptosis, and represses mitochondrial oxygen consumption. J Biol Chem 288(36):25964–25975. 23902766 10.1074/jbc.M113.459784 PMC3764801

[B59] Hiemstra PS, McCray PB Jr, Bals R. 2015. The innate immune function of airway epithelial cells in inflammatory lung disease. Eur Respir J 45(4):1150–1162. 25700381 10.1183/09031936.00141514 PMC4719567

[B60] Hou W, Jin YH, Kang HS, Kim BS. 2014. Interleukin-6 (IL-6) and IL-17 synergistically promote viral persistence by inhibiting cellular apoptosis and cytotoxic T cell function. J Virol 88(15):8479–8489. 24829345 10.1128/JVI.00724-14 PMC4135960

[B61] Huang C, Wang Y, Li X, Ren L, Zhao J, Hu Y, Zhang L, Fan G, Xu J, Gu X, Cheng Z, Yu T, Xia J, Wei Y, Wu W, Xie X, Yin W, Li H, Liu M, Xiao Y, Gao H, Guo L, Xie J, Wang G, Jiang R, Gao Z, Jin Q, Wang J, Cao B. 2020. Clinical features of patients infected with 2019 novel coronavirus in Wuhan, China. Lancet 395(10223):497–506. 31986264 10.1016/S0140-6736(20)30183-5 PMC7159299

[B62] Hunninghake GM, Cho MH, Tesfaigzi Y, Soto-Quiros ME, Avila L, Lasky-Su J, Stidley C, Melén E, Söderhäll C, Hallberg J, Kull I, Kere J, Svartengren M, Pershagen G, Wickman M, Lange C, Demeo DL, Hersh CP, Klanderman BJ, Raby BA, Sparrow D, Shapiro SD, Silverman EK, Litonjua AA, Weiss ST, Celedón JC. 2009. MMP12, lung function, and COPD in high-risk populations. N Engl J Med 361(27):2599–2608. 20018959 10.1056/NEJMoa0904006 PMC2904064

[B63] Ichikado K, Muranaka H, Gushima Y, Kotani T, Nader HM, Fujimoto K, Johkoh T, Iwamoto N, Kawamura K, Nagano J, Fukuda K, Hirata N, Yoshinaga T, Ichiyasu H, Tsumura S, Kohrogi H, Kawaguchi A, Yoshioka M, Sakuma T, Suga M. 2012. Fibroproliferative changes on high-resolution CT in the acute respiratory distress syndrome predict mortality and ventilator dependency: a prospective observational cohort study. BMJ 2(2):e000545. 10.1136/bmjopen-2011-000545 PMC3293132 22382117

[B64] Johns Hopkins Coronavirus Resource Center. 2022. Available at: https://coronavirus.jhu.edu/

[B65] Johnson JR, Nishioka M, Chakir J, Risse PA, Almaghlouth I, Bazarbashi AN, Plante S, Martin JG, Eidelman D, Hamid Q. 2013. IL-22 contributes to TGF-β1-mediated epithelial-mesenchymal transition in asthmatic bronchial epithelial cells. Respir Res 14(1):118. 24283210 10.1186/1465-9921-14-118 PMC4176096

[B66] Karamanos NK, Theocharis AD, Piperigkou Z, Manou D, Passi A, Skandalis SS, Vynios DH, Orian-Rousseau V, Ricard-Blum S, Schmelzer CEH, Duca L, Durbee M, Afratis NA, Troeberg L, Franchi M, Masola V, Onisto M. 2021. A guide to the composition and functions of the extracellular matrix. FEBS J 288(24):6850–6912. 33605520 10.1111/febs.15776

[B67] Karsdal MA, Nielsen S, Leeming DJ, Langholm LL, Nielsen MJ, Manon-Jensen T, Siebuhr A, Gudmann NS, Rønnow S, Sand JM, Daniels SJ, Mortensen JH, Schuppan D. 2017. The good and the bad collagens of fibrosis—their role in signaling and organ function. Adv Drug Deliv Rev 121:43–56. 28736303 10.1016/j.addr.2017.07.014

[B68] Kaźmierczak-Siedlecka K, Vitale E, Makarewicz W. 2020. COVID-19—gastrointestinal and gut microbiota-related aspects. Eur Rev Med Pharmacol Sci 24(20):10853–10859. 33155247 10.26355/eurrev_202010_23448

[B69] Klok FA, Kruip M, van der Meer NJM, Arbous MS, Gommers DAMPJ, Kant KM, Kaptein FHJ, van Paassen J, Stals MAM, Huisman MV, Endeman H. 2020. Incidence of thrombotic complications in critically ill ICU patients with COVID-19. Thromb Res 191:145–147. 32291094 10.1016/j.thromres.2020.04.013 PMC7146714

[B70] Kobayashi T, Tanaka K, Fujita T, Umezawa H, Amano H, Yoshioka K, Naito Y, Hatano M, Kimura S, Tatsumi K, Kasuya Y. 2015. Bidirectional role of IL-6 signal in pathogenesis of lung fibrosis. Respir Res 16(1):99. 26289430 10.1186/s12931-015-0261-z PMC4546032

[B71] Konda M, Dodda B, Konala VM, Naramala S, Adapa S. 2020. Potential zoonotic origins of SARS-CoV-2 and insights for preventing future pandemics through one health approach. Cureus 12(6):e8932. 32760632 10.7759/cureus.8932 PMC7392364

[B72] Kong Y, Han J, Wu X, Zeng H, Liu J, Zhang H. 2020. VEGF-D: a novel biomarker for detection of COVID-19 progression. Crit Care 24(1):373. 32576222 10.1186/s13054-020-03079-y PMC7309201

[B73] Kopf M, Baumann H, Freer G, Freudenberg M, Lamers M, Kishimoto T, Zinkernagel R, Bluethmann H, Köhler G. 1994. Impaired immune and acute-phase responses in interleukin-6-deficient mice. Nature 368(6469):339–342. 8127368 10.1038/368339a0

[B74] Kuo TM, Hu CP, Chen YL, Hong MH, Jeng KS, Liang CC, Chen ML, Chang C. 2009. HBV replication is significantly reduced by IL-6. J Biomed Sci 16(1):41. 19374779 10.1186/1423-0127-16-41 PMC2687430

[B75] Lagente V, Manoury B, Néan S, Le Quément C, Martin-Chouly C, Boichot E. 2005. Role of matrix metalloproteinases in the development of airway inflammation and remodeling. Braz J Med Biol Res 38:1521–1530. 16172745 10.1590/s0100-879x2005001000009

[B76] Lan S-H, Lai C-C, Huang H-T, Chang S-P, Lu L-C, Hsueh P-R. 2020. Tocilizumab for severe COVID-19: a systematic review and meta-analysis. Int J Antimicrob Agents 56(3):106103. 32712333 10.1016/j.ijantimicag.2020.106103 PMC7377685

[B77] Lancaster LH, de Andrade JA, Zibrak JD, Padilla ML, Albera C, Nathan SD, Wijsenbeek MS, Stauffer JL, Kirchgaessler KU, Costabel U. 2017. Pirfenidone safety and adverse event management in idiopathic pulmonary fibrosis. Eur Respir Rev 26(146):170057. 29212837 10.1183/16000617.0057-2017 PMC9488585

[B78] Lauder SN, Jones E, Smart K, Bloom A, Williams AS, Hindley JP, Ondondo B, Taylor PR, Clement M, Fielding C, Godkin AJ, Jones SA, Gallimore AM. 2013. Interleukin-6 limits influenza-induced inflammation and protects against fatal lung pathology. Eur J Immunol 43(10):2613–2625. 23857287 10.1002/eji.201243018 PMC3886386

[B79] Lee Y, Awasthi A, Yosef N, Quintana FJ, Xiao S, Peters A, Wu C, Kleinewietfeld M, Kunder S, Hafler DA, Sobel RA, Regev A, Kuchroo VK. 2012. Induction and molecular signature of pathogenic TH17 cells. Nat Immunol 13(10):991–999. 22961052 10.1038/ni.2416 PMC3459594

[B80] Levi M, van der Poll T. 2005. Two-way interactions between inflammation and coagulation. Trends Cardiovasc Med 15(7):254–259. 16226680 10.1016/j.tcm.2005.07.004

[B81] Li F. 2016. Structure, function, and evolution of coronavirus spike proteins. Annu Rev Virol 3(1):237–261. 27578435 10.1146/annurev-virology-110615-042301 PMC5457962

[B82] Li MO, Wan YY, Sanjabi S, Robertson AK, Flavell RA. 2006. Transforming growth factor-beta regulation of immune responses. Annu Rev Immunol 9924:99–146. 10.1146/annurev.immunol.24.021605.090737 16551245

[B83] Li S-W, Wang C-Y, Jou Y-J, Yang T-C, Huang S-H, Wan L, Lin Y-J, Lin C-W. 2016. SARS coronavirus papain-like protease induces Egr-1-dependent up-regulation of TGF-β1 via ROS/p38 MAPK/STAT3 pathway. Sci Rep 6(1):25754. 27173006 10.1038/srep25754 PMC4865725

[B84] Li X, Geng M, Peng Y, Meng L, Lu S. 2020. Molecular immune pathogenesis and diagnosis of COVID-19. J Pharm Anal 10(2):102–108. 32282863 10.1016/j.jpha.2020.03.001 PMC7104082

[B85] Liao M, Liu Y, Yuan J, Wen Y, Xu G, Zhao J, Cheng L, Li J, Wang X, Wang F, Liu L, Amit I, Zhang S, Zhang Z. 2020. Single-cell landscape of bronchoalveolar immune cells in patients with COVID-19. Nat Med 26(6):842–844. 32398875 10.1038/s41591-020-0901-9

[B86] Liu J, Khalil RA. 2017. Matrix metalloproteinase inhibitors as investigational and therapeutic tools in unrestrained tissue remodeling and pathological disorders. Prog Mol Biol Transl Sci 148:355–420. 28662828 10.1016/bs.pmbts.2017.04.003 PMC5548434

[B87] Lu L, Zhang H, Dauphars DJ, He YW. 2021. A potential role of interleukin 10 in COVID-19 pathogenesis. Trends Immunol 42(1):3–5. 33214057 10.1016/j.it.2020.10.012 PMC7605819

[B88] Maione F, Casillo GM, Raucci F, Salvatore C, Ambrosini G, Costa L, Scarpa R, Caso F, Bucci M. 2021. Interleukin-17A (IL-17A): a silent amplifier of COVID-19. Biomed Pharmacother 142:111980. 34364043 10.1016/j.biopha.2021.111980 PMC8318692

[B89] Marten NW, Zhou J. 2005. The role of metalloproteinases in corona virus infection. In: Lavi E, Constantinescu CS, eds. Experimental models of multiple sclerosis. Los Angeles, CA: Springer Science+Business Media. pp 839–848.

[B90] Mason RJ. 2020. Pathogenesis of COVID-19 from a cell biology perspective. Eur Respir J 55(4):2000607. 32269085 10.1183/13993003.00607-2020 PMC7144260

[B91] Matthay MA, Zemans RL, Zimmerman GA, Arabi YM, Beitler JR, Mercat A, Herridge M, Randolph AG, Calfee CS. 2019. Acute respiratory distress syndrome. Nat Rev Dis Primers 5(1):18. 30872586 10.1038/s41572-019-0069-0 PMC6709677

[B92] Meng J, Ma Y, Jia J, Wang M, Teng J, Shi H, Liu H, Su Y, Ye J, Sun Y, Cheng X, Chi H, Liu T, Zhu D, Zhou Z, Wan L, Wang Z, Wang F, Qiao X, Chen X, Zhang H, Tang Z, Yang C, Hu Q. 2021. Cytokine storm in coronavirus disease 2019 and adult-onset still's disease: similarities and differences. Front Immunol 11:603389. 33552062 10.3389/fimmu.2020.603389 PMC7856388

[B93] Merad M, Martin JC. 2020. Pathological inflammation in patients with COVID-19: a key role for monocytes and macrophages. Nat Rev Immunol 20(6):355–362. 32376901 10.1038/s41577-020-0331-4 PMC7201395

[B94] Montalvo Villalba MC, Ramírez OV, Muné Jiménez M, Arencibia Garcia A, Martinez Alfonso J, González Baéz G, Roque Arrieta R, Rosell Simón D, Alvárez Gainza D, Sierra Vázquez B, Resik Aguirre S, Guzmán Tirado MG. 2020. Interferon gamma, TGF-β1 and RANTES expression in upper airway samples from SARS-CoV-2 infected patients. Clin Immunol 220:108576. 32866645 10.1016/j.clim.2020.108576 PMC7455570

[B95] Morrell ED, Mikacenic C, Gong KQ, Kosamo S, Wurfel MM, Manicone AM. 2020. Alveolar MMP28 is associated with clinical outcomes and measures of lung injury in acute respiratory distress syndrome. Crit Care 24(1):141. 32268921 10.1186/s13054-020-02847-0 PMC7144344

[B96] Munger JS, Huang X, Kawakatsu H, Griffiths MJ, Dalton SL, Wu J, Pittet JF, Kaminski N, Garat C, Matthay MA, Rifkin DB, Sheppard D. 1999. The integrin alpha v beta 6 binds and activates latent TGF beta 1: a mechanism for regulating pulmonary inflammation and fibrosis. Cell 96(3):319–328. 10025398 10.1016/s0092-8674(00)80545-0

[B97] O'Kane CM, McKeown SW, Perkins GD, Bassford CR, Gao F, Thickett DR, McAuley DF. 2009. Salbutamol up-regulates matrix metalloproteinase-9 in the alveolar space in the acute respiratory distress syndrome. Crit Care Med 37(7):2242–2249. 19487934 10.1097/CCM.0b013e3181a5506c

[B98] Ong CH, Tham CL, Harith HH, Firdaus N, Israf DA. 2021. TGF-β-induced fibrosis: a review on the underlying mechanism and potential therapeutic strategies. Eur J Pharmacol 911:174510. 34560077 10.1016/j.ejphar.2021.174510

[B99] Ooi GC, Khong PL, Müller NL, Yiu WC, Zhou LJ, Ho JC, Lam B, Nicolaou S, Tsang KW. 2004. Severe acute respiratory syndrome: temporal lung changes at thin-section CT in 30 patients. Radiology 230(3):836–844. 14990845 10.1148/radiol.2303030853

[B100] Pala D, Pistis M. 2021. Anti-IL5 Drugs in COVID-19 patients: role of eosinophils in SARS-CoV-2-induced immunopathology. Front Pharmacol 12:622554. 33767626 10.3389/fphar.2021.622554 PMC7985166

[B101] Pandolfi L, Bozzini S, Frangipane V, Percivalle E, De Luigi A, Violatto MB, Lopez G, Gabanti E, Carsana L, D'Amato M, Morosini M, De Amici M, Nebuloni M, Fossali T, Colombo R, Saracino L, Codullo V, Gnecchi M, Bigini P, Baldanti F, Lilleri D, Meloni F. 2021. Neutrophil extracellular traps induce the epithelial-mesenchymal transition: implications in post-COVID-19 fibrosis. Front Immunol 12:663303. 34194429 10.3389/fimmu.2021.663303 PMC8236949

[B102] Perisetti A, Gajendran M, Mann R, Elhanafi S, Goyal H. 2020. COVID-19 extrapulmonary illness—special gastrointestinal and hepatic considerations. Dis Mon 66(9):101064. 32807535 10.1016/j.disamonth.2020.101064 PMC7386425

[B103] Planté-Bordeneuve T, Pilette C, Froidure A. 2021. The epithelial-immune crosstalk in pulmonary fibrosis. Front Immunol 12:631235. 34093523 10.3389/fimmu.2021.631235 PMC8170303

[B104] Potere N, Batticciotto A, Vecchié A, Porreca E, Cappelli A, Abbate A, Dentali F, Bonaventura A. 2021. The role of IL-6 and IL-6 blockade in COVID-19. Expert Rev Clin Immunol 17(6):601–618. 33874829 10.1080/1744666X.2021.1919086

[B105] Qin C, Zhou L, Hu Z, Zhang S, Yang S, Tao Y, Xie C, Ma K, Shang K, Wang W, Tian DS. 2020. Dysregulation of immune response in patients with coronavirus 2019 (COVID-19) in Wuhan, China. Clin Infect Dis 17(15):762–768. 10.1093/cid/ciaa248 PMC7108125 32161940

[B106] Quintanilla M, del Castillo G, Kocic J, Santibañez JF. 2012. TGF-β and MMPs: A complex regulatory loop involved in tumor progression. In: Oshiro N, Miyagi E, eds. Matrix Metalloproteinases: Biology, Functions and Clinical Implications. UK: Nova Science Publishers. pp 1–37.

[B107] Rajah R, Katz L, Nunn S, Solberg P, Beers T, Cohen P. 1995. Insulin-like growth factor binding protein (IGFBP) proteases: functional regulators of cell growth. Prog Growth Factor Res 6(2–4):273–284. 8817670 10.1016/0955-2235(95)00012-7

[B108] Ramachandran P, Dobie R, Wilson-Kanamori JR, Dora EF, Henderson BEP, Luu NT, Portman JR, Matchett KP, Brice M, Marwick JA, Taylor RS, Efremova M, Vento-Tormo R, Carragher NO, Kendall TJ, Fallowfield JA, Harrison EM, Mole DJ, Wigmore SJ, Newsome PN, Weston CJ, Iredale JP, Tacke F, Pollard JW, Ponting CP, Marioni JC, Teichmann SA, Henderson NC. 2019. Resolving the fibrotic niche of human liver cirrhosis at single-cell level. Nature 575(7783):512–518. 31597160 10.1038/s41586-019-1631-3 PMC6876711

[B109] Rao S, Lau A, So HC. 2020. Exploring diseases/traits and blood proteins causally related to expression of ACE2, the putative receptor of SARS-CoV-2: a Mendelian randomization analysis highlights tentative relevance of diabetes-related traits. Diabetes Care 44(7):1416–1426. 10.2337/dc20-0643 32430459

[B110] Roan F, Obata-Ninomiya K, Ziegler SF. 2019. Epithelial cell-derived cytokines: more than just signaling the alarm. J Clin Invest 129(4):1441–1451. 30932910 10.1172/JCI124606 PMC6436879

[B111] Rogliani P, Calzetta L, Coppola A, Puxeddu E, Sergiacomi G, D'Amato D, Orlacchio A. 2020. Are there pulmonary sequelae in patients recovering from COVID-19? Respir Res 21(1):286. 33126869 10.1186/s12931-020-01550-6 PMC7598236

[B112] Rosas IO, Richards TJ, Konishi K, Zhang Y, Gibson K, Lokshin AE, Lindell KO, Cisneros J, Macdonald SD, Pardo A, Sciurba F, Dauber J, Selman M, Gochuico BR, Kaminski N. 2008. MMP1 and MMP7 as potential peripheral blood biomarkers in idiopathic pulmonary fibrosis. PLoS Med 5(4):e93. 18447576 10.1371/journal.pmed.0050093 PMC2346504

[B113] Ruscitti P, Berardicurti O, Barile A, Cipriani P, Shoenfeld Y, Iagnocco A, Giacomelli R. 2020. Severe COVID-19 and related hyperferritinaemia: more than an innocent bystander? Ann Rheum Dis 79(11):1515–1516. 32434816 10.1136/annrheumdis-2020-217618

[B114] Sadeghi A, Tahmasebi S, Mahmood A, Kuznetsova M, Valizadeh H, Taghizadieh A, Nazemiyeh M, Aghebati-Maleki L, Jadidi-Niaragh F, Abbaspour-Aghdam S, Roshangar L, Mikaeili H, Ahmadi M. 2021. Th17 and Treg cells function in SARS-CoV2 patients compared with healthy controls. J Cell Physiol 236(4):2829–2839. 32926425 10.1002/jcp.30047

[B115] Safont B, Tarraso J, Rodriguez-Borja E, Fernández-Fabrellas E, Sancho-Chust JN, Molina V, Lopez-Ramirez C, Lope-Martinez A, Cabanes L, Andreu AL, Herrera S, Lahosa C, Ros JA, Rodriguez-Hermosa JL, Soriano JB, Moret-Tatay I, Carbonell-Asins JA, Mulet A, Signes-Costa J. 2022. Lung function, radiological findings and biomarkers of fibrogenesis in a cohort of COVID-19 patients six months after hospital discharge. Arch Bronconeumol 58(2):142–149. 34497426 10.1016/j.arbres.2021.08.014 PMC8414844

[B116] Sahu T, Mehta A, Ratre YK, Jaiswal A, Vishvakarma NK, Bhaskar LVKS, Verma HK. 2021. Current understanding of the impact of COVID-19 on gastrointestinal disease: challenges and openings. World J Gastroenterol 27(6):449–469. 33642821 10.3748/wjg.v27.i6.449 PMC7896435

[B117] Saito A, Horie M, Nagase T. 2018. TGF-β signaling in lung health and disease. Int J Mol Sci 19(8):460. 10.3390/ijms19082460 PMC6121238 30127261

[B118] Schultz-Cherry S, Hinshaw VS. 1996. Influenza virus neuraminidase activates latent transforming growth factor beta. Journal of virology 70(12):8624–8629. 8970987 10.1128/jvi.70.12.8624-8629.1996 PMC190955

[B119] Selman M, King TE, Pardo A. 2001. Idiopathic pulmonary fibrosis: prevailing and evolving hypotheses about its pathogenesis and implications for therapy. Ann Intern Med 134(2):136–151. 11177318 10.7326/0003-4819-134-2-200101160-00015

[B120] Selman M, Pardo A. 2020. The leading role of epithelial cells in the pathogenesis of idiopathic pulmonary fibrosis. Cell Signal 66:109482. 31760172 10.1016/j.cellsig.2019.109482

[B121] Shang J, Wan Y, Luo C, Ye G, Geng Q, Auerbach A, Li F. 2020. Cell entry mechanisms of SARS-CoV-2. Proc Natl Acad Sci U S A 117(21):11727–11734. 32376634 10.1073/pnas.2003138117 PMC7260975

[B122] Shi H, Wang W, Yin J, Ouyang Y, Pang L, Feng Y, Qiao L, Guo X, Shi H, Jin R, Chen D. 2020a. The inhibition of IL-2/IL-2R gives rise to CD8^+^ T cell and lymphocyte decrease through JAK1 STAT5 in critical patients with COVID-19 pneumonia. Cell Death Dis. 11(6):429. 32513989 10.1038/s41419-020-2636-4 PMC7276960

[B123] Shi M, Zhu J, Wang R, Chen X, Mi L, Walz T, Springer TA. 2011. Latent TGF-β structure and activation. Nature 474(7351):343–349. 21677751 10.1038/nature10152 PMC4717672

[B124] Shi S, Su M, Shen G, Hu Y, Yi F, Zeng Z, Zhu P, Yang G, Zhou H, Li Q, Xie X. 2020b. Matrix metalloproteinase 3 as a valuable marker for patients with COVID-19. J Med Virol 10:1002. 10.1002/jmv.26235 PMC7362036 32603484

[B125] Shiomi T, Lemaître V, D'Armiento J, Okada Y. 2010. Matrix metalloproteinases, a disintegrin and metalloproteinases, and a disintegrin and metalloproteinases with thrombospondin motifs in non-neoplastic diseases. Pathol Int 60(7):477–496. 20594269 10.1111/j.1440-1827.2010.02547.x PMC3745773

[B126] Takeuchi O, Akira S. 2010. Pattern recognition receptors and inflammation. Cell 40(6):805–820. 10.1016/j.cell.2010.01.022 20303872

[B127] Tang D, Comish P, Kang R. 2020a. The hallmarks of COVID-19 disease. PLoS Pathog 15(5):e1008536. 10.1371/journal.ppat.1008536 PMC7244094 32442210

[B128] Tang N, Li D, Wang X, Sun Z. 2020b. Abnormal coagulation parameters are associated with poor prognosis in patients with novel coronavirus pneumonia. J Thromb Haemost 18(4):844–847. 32073213 10.1111/jth.14768 PMC7166509

[B129] Tang Y, Liu J, Zhang D, Xu Z, Ji J, Wen C. 2020c. Cytokine storm in COVID-19: the current evidence and treatment strategies. Front Immunol 11:1708. 32754163 10.3389/fimmu.2020.01708 PMC7365923

[B130] Thomas BJ, Kan-O K, Loveland KL, Elias JA, Bardin PG. 2016. In the shadow of fibrosis: innate immune suppression mediated by transforming growth factor-β. Am J Respir Cell Mol Biol 55(6):759–766. 27603223 10.1165/rcmb.2016-0248PS

[B131] Toki S, Goleniewska K, Zhang J, Zhou W, Newcomb DC, Zhou B, Kita H, Boyd KL, Peebles RS Jr. 2020. TSLP and IL-33 reciprocally promote each other's lung protein expression and ILC2 receptor expression to enhance innate type-2 airway inflammation. Allergy 75(7):1606–1617. 31975538 10.1111/all.14196 PMC7354889

[B132] Trojanek JB, Cobos-Correa A, Diemer S, Kormann M, Schubert SC, Zhou-Suckow Z, Agrawal R, Duerr J, Wagner CJ, Schatterny J, Hirtz S, Sommerburg O, Hartl D, Schultz C, Mall MA. 2014. Airway mucus obstruction triggers macrophage activation and matrix metalloproteinase 12-dependent emphysema. Am J Respir Cell Mol Biol 51(5):709–720. 24828142 10.1165/rcmb.2013-0407OC

[B133] Ueland T, Holter JC, Holten AR, Müller KE, Lind A, Bekken GK, Dudman S, Aukrust P, Dyrhol-Riise AM, Heggelund L. 2020. Distinct and early increase in circulating MMP-9 in COVID-19 patients with respiratory failure. J Infect 81(3):e41–e43. 32603675 10.1016/j.jinf.2020.06.061 PMC7320854

[B134] van de Veerdonk FL, Netea MG. 2020. Blocking IL-1 to prevent respiratory failure in COVID-19. Crit Care 24(1):445. 32682440 10.1186/s13054-020-03166-0 PMC7411343

[B135] Vaz de Paula CB, de Azevedo MLV, Nagashima S, Martins APC, Malaquias MAS, Miggiolaro AFRDS, da Silva Motta Júnior J, Avelino G, do Carmo LAP, Carstens LB, de Noronha L. 2020. IL-4/IL-13 remodeling pathway of COVID-19 lung injury. Sci Rep 10(1):18689. 33122784 10.1038/s41598-020-75659-5 PMC7596721

[B136] Villapol S. 2020. Gastrointestinal symptoms associated with COVID-19: impact on the gut microbiome. Transl Res 226:57–69. 32827705 10.1016/j.trsl.2020.08.004 PMC7438210

[B137] Wan J, Zhang G, Li X, Qiu X, Ouyang J, Dai J, Min S. 2021. Matrix metalloproteinase 3: a promoting and destabilizing factor in the pathogenesis of disease and cell differentiation. Front Physiol 12:663978. 34276395 10.3389/fphys.2021.663978 PMC8283010

[B138] Wang D, Hu B, Hu C, Zhu F, Liu X, Zhang J, Wang B, Xiang H, Cheng Z, Xiong Y, Zhao Y, Li Y, Wang X, Peng Z. 2020a. Clinical characteristics of 138 hospitalized patients with 2019 novel coronavirus-infected pneumonia in Wuhan, China. JAMA 323(11):1061–1069. 32031570 10.1001/jama.2020.1585 PMC7042881

[B139] Wang F, Nie J, Wang H, Zhao Q, Xiong Y, Deng L, Song S, Ma Z, Mo P, Zhang Y. 2020b. Characteristics of peripheral lymphocyte subset alteration in COVID-19 pneumonia. J Infect Dis 221(11):1762–1769. 32227123 10.1093/infdis/jiaa150 PMC7184346

[B140] Warner RL, Lewis CS, Beltran L, Younkin EM, Varani J, Johnson KJ. 2001. The role of metalloelastase in immune complex-induced acute lung injury. Am J Pathol 158(6):2139–2144. 11395391 10.1016/S0002-9440(10)64685-8 PMC1892006

[B141] Weisberg SP, Connors TJ, Zhu Y, Baldwin MR, Lin WH, Wontakal S, Szabo PA, Wells SB, Dogra P, Gray J, Idzikowski E, Stelitano D, Bovier FT, Davis-Porada J, Matsumoto R, Poon MML, Chait M, Mathieu C, Horvat B, Decimo D, Hudson KE, Zotti FD, Bitan ZC, La Carpia F, Ferrara SA, Mace E, Milner J, Moscona A, Hod E, Porotto M, Farber DL. 2021. Distinct antibody responses to SARS-CoV-2 in children and adults across the COVID-19 clinical spectrum. Nat Immunol 22(1):25–31. 33154590 10.1038/s41590-020-00826-9 PMC8136619

[B142] Wilson EB, Livingstone AM. 2008. Cutting edge: CD4^+^ T cell-derived IL-2 is essential for help-dependent primary CD8^+^ T cell responses. J Immunol 181(11):7445–7448. 19017930 10.4049/jimmunol.181.11.7445

[B143] Witkowski M, Tizian C, Ferreira-Gomes M, Niemeyer D, Jones TC, Heinrich F, Frischbutter S, Angermair S, Hohnstein T, Mattiola I, Nawrath P, McEwen S, Zocche S, Viviano E, Heinz GA, Maurer M, Kölsch U, Chua RL, Aschman T, Meisel C, Radke J, Sawitzki B, Roehmel J, Allers K, Moos V, Schneider T, Hanitsch L, Mall MA, Conrad C, Radbruch H, Duerr CU, Trapani JA, Marcenaro E, Kallinich T, Corman VM, Kurth F, Sander LE, Drosten C, Treskatsch S, Durek P, Kruglov A, Radbruch A, Mashreghi MF, Diefenbach A. 2021. Untimely TGFβ responses in COVID-19 limit antiviral functions of NK cells. Nature 600(7888):295–301. 34695836 10.1038/s41586-021-04142-6

[B144] Woolf SH, Chapman DA, Lee JH. 2021. COVID-19 as the leading cause of death in the United States. JAMA 12(325):123–124. 10.1001/jama.2020.24865 PMC8553021 33331845

[B145] Wu W, Dietze KK, Gibbert K, Lang KS, Trilling M, Yan H, Wu J, Yang D, Lu M, Roggendorf M, Dittmer U, Liu J. 2015. TLR ligand induced IL-6 counter-regulates the anti-viral CD8(+) T cell response during an acute retrovirus infection. Sci Rep 5:10501. 25994622 10.1038/srep10501 PMC4440206

[B146] Wu Y, Zou F, Lu Y, Li X, Li F, Feng X, Sun X, Liu Y. 2019. SETD7 promotes TNF-α-induced proliferation and migration of airway smooth muscle cells in vitro through enhancing NF-κB/CD38 signaling. Int Immunopharmacol 72:459–466. 31035088 10.1016/j.intimp.2019.04.043

[B147] Xing ZH, Sun X, Xu L, Wu Q, Li L, Wu XJ, Shao XG, Zhao XQ, Wang JH, Ma LY, Wang K. 2015. Thin-section computed tomography detects long-term pulmonary sequelae 3 years after novel influenza A virus-associated pneumonia. Chin Med J (Engl) 128(5):902–908. 25836610 10.4103/0366-6999.154285 PMC4834006

[B148] Xu YH, Dong JH, An WM, Lv XY, Yin XP, Zhang JZ, Dong L, Ma X, Zhang HJ, Gao BL. 2020a. Clinical and computed tomographic imaging features of novel coronavirus pneumonia caused by SARS-CoV-2. J Infect 80(4):394–400. 32109443 10.1016/j.jinf.2020.02.017 PMC7102535

[B149] Xu Z, Shi L, Wang Y, Zhang J, Huang L, Zhang C, Liu S, Zhao P, Liu H, Zhu L, Tai Y, Bai C, Gao T, Song J, Xia P, Dong J, Zhao J, Wang FS. 2020b. Pathological findings of COVID-19 associated with acute respiratory distress syndrome. Lancet 8(4):420–422. 10.1016/S2213-2600(20)30076-X PMC7164771 32085846

[B150] Xu ZS, Shu T, Kang L, Wu D, Zhou X, Liao BW, Sun XL, Zhou X, Wang YY. 2020c. Temporal profiling of plasma cytokines, chemokines and growth factors from mild, severe and fatal COVID-19 patients. Signal Transduct Target Ther 5(1):100. 32561706 10.1038/s41392-020-0211-1 PMC7303571

[B151] Yalçın Kehribar D, Cihangiroğlu M, Sehmen E, Avcı B, Çapraz M, Boran M, Günaydin C, Özgen M. 2020. The assessment of the serum levels of TWEAK and prostaglandin F2α in COVID–19. Turk J Med Sci 50(8):1786–1791. 32979900 10.3906/sag-2006-96 PMC7778458

[B152] Yamashita CM, Dolgonos L, Zemans RL, Young SK, Robertson J, Briones N, Suzuki T, Campbell MN, Gauldie J, Radisky DC, Riches DW, Yu G, Kaminski N, McCulloch CAG, Downey GP. 2011. Matrix metalloproteinase 3 is a mediator of pulmonary fibrosis. Am J Pathol 79(4):1733–1745. 10.1016/j.ajpath.2011.06.041 PMC3181358 21871427

[B153] Yang J, Pan X, Wang L, Yu G. 2020. Alveolar cells under mechanical stressed niche: critical contributors to pulmonary fibrosis. Mol Med 26(1):95. 33054759 10.1186/s10020-020-00223-w PMC7556585

[B154] Yang ML, Wang CT, Yang SJ, Leu CH, Chen SH, Wu CL, Shiau AL. 2017. IL-6 ameliorates acute lung injury in influenza virus infection. Sci Rep 6(7):43829. 10.1038/srep43829 PMC5338329 28262742

[B155] Zhang F, Mears JR, Shakib L, Beynor JI, Shanaj S, Korsunsky I, Nathan A; Accelerating Medicines Partnership Rheumatoid Arthritis and Systemic Lupus Erythematosus (AMP RA/SLE) Consortium; Donlin LT, Raychaudhuri S. 2021. IFN-γ andTNF-α drive a CXCL10^+^ CCL2^+^ macrophage phenotype expanded in severe COVID-19 lungs and inflammatory diseases with tissue inflammation. Genome Med 13(1):64. 33879239 10.1186/s13073-021-00881-3 PMC8057009

[B156] Zhou Y, Fu B, Zheng X, Wang D, Zhao C, Qi Y, Sun R, Tian Z, Xu X, Wei H. 2020. Pathogenic T-cells and inflammatory monocytes incite inflammatory storms in severe COVID-19 patients. Natl Sci Rev 7(6):998–1002. 34676125 10.1093/nsr/nwaa041 PMC7108005

